# Selective growth of Ti^3+^/TiO_2_/CNT and Ti^3+^/TiO_2_/C nanocomposite for enhanced visible-light utilization to degrade organic pollutants by lowering TiO_2_-bandgap

**DOI:** 10.1038/s41598-021-89026-5

**Published:** 2021-05-04

**Authors:** Jeasmin Akter, Md. Abu Hanif, Md. Akherul Islam, Kamal Prasad Sapkota, Jae Ryang Hahn

**Affiliations:** 1 Department of Chemistry, Jeonbuk National University, Jeonju, 54896 Korea; 2 Department of Bioactive Material Sciences, Jeonbuk National University, Jeonju, 54896 Korea; 3 Textile Engineering, Chemistry and Science, North Carolina State University, 2401 Research Dr., Raleigh, NC 27695 USA

**Keywords:** Photocatalysis, Photocatalysis

## Abstract

A convenient route was developed for the selective preparation of two stable nanocomposites, Ti^3+^/TiO_2_/CNT (labeled as TTOC-1 and TTOC-3) and Ti^3+^/TiO_2_/carbon layer (labeled as TTOC-2), from the same precursor by varying the amount of single-walled carbon nanotubes used in the synthesis. TiO_2_ is an effective photocatalyst; however, its wide bandgap limits its usefulness to the UV region. As a solution to this problem, our prepared nanocomposites exhibit a small bandgap and wide visible-light (VL) absorption because of the introduction of carbonaceous species and Ti^3+^ vacancies. The photocatalytic efficiency of the nanocomposites was examined via the degradation of methylene blue dye under VL. Excellent photocatalytic activity of 83%, 98%, and 93% was observed for TTOC-1, TTOC-2, and TTOC-3 nanocomposites within 25 min. In addition, the photocatalytic degradation efficiency of TTOC-2 toward methyl orange, phenol, rhodamine B, and congo red was 28%, 69%, 71%, and 91%, respectively, under similar experimental conditions after 25 min. Higher reusability and structural integrity of the as-synthesized photocatalyst were confirmed within five consecutive runs by photocatalytic test and X-ray diffraction analysis, respectively. The resulting nanocomposites provide new insights into the development of VL-active and stable photocatalysts with high efficiencies.

## Introduction

Environmental pollution problems are constantly increasing because of the rapidly expanding world population and increasing industrialization. Approximately 1 × 10^5^ forms of dyes are produced with a yearly rate greater than 1 × 10^6^ tons; these dyes are used in various industries such as the leather, textile, printing, paper, pigment, paint, plastic, and rubber industries^1–3^. A huge amount of effluent is released throughout dyeing processes into water bodies and the surrounding environment. Dye pollutants can cause dermatitis, allergy, cancer, kidney dysfunction, skin irritation, and problems with the reproductive system and liver in humans^4–7^. Several dyes such as methylene blue (MB), congo red (CR), rhodamine B (RhB), and colorless compound phenol has been used extensively in various fields. Their function is very important in the healthcare and industries sector. However, wastewater contaminated by such organic dyes causes several human health and environmental problems, making the development and implementation of an efficient and green technique to resolve this problem urgent^4–10^.

Numerous research groups are working to efficiently solve environmental problems related to water resources. Various approaches have been developed to eliminate dyes from industrial wastewater and water, including adsorption, electrolysis, ion exchange, conventional coagulation, chemical precipitation, and photocatalytic degradation^4,11,12^. Among the methods for the elimination of dyes, photocatalytic degradation and adsorption are acknowledged as efficient, inexpensive, and environmentally friendly techniques. However, despite the cost-effectiveness of adsorption materials used for dye removal, the adsorption process produces large amounts of solid wastes.

Photocatalytic degradation of organic pollutants is advantageous because of its eco-friendly, safety, and low cost^11–15^. Semiconductor photocatalysts have attracted intensive attention because of their potential applications in dye-sensitized solar cells, pollutant degradation, biocatalysis, and photocatalytic hydrogen evolution. In particular, titanium dioxide (TiO_2_) nanomaterials have been commonly studied because of their low toxicity, superior photocatalytic activity, and good chemical and biological stability. However, two significant shortcomings of TiO_2_ restrict its application to the UV region: its relatively wide bandgap (3.0–3.2 eV), and the rapid recombination of photoexcited electrons (e^−^) and holes (h^+^) in its lattice^11–14,16,17^.

As a result, the development of new TiO_2_ photocatalytic systems with enhanced visible-light (VL) absorption is critical and a formidable challenge^18^. To date, several techniques have been used to prolong the separation lifetime of e^−^/h^+^ pairs and improve the VL absorption of TiO_2_. Among them, heteroelement doping is an excellent approach to addressing these challenges. Cations such as Fe^3+^, Mn^3+^, V^4+^, Re^5+^, Os^3+^, Mo^4+^, and Rh^3+^ have been used as dopants in TiO_2_^11,14,19^. Doping of nonmetals, resulting in F/TiO_2_, S/TiO_2_, N/TiO_2_, C/TiO_2_, and B/TiO_2_, has also been reported^14,20^. However, thermal instability and the likelihood of charge recombination both increase with the introduction of heteroelements. To overcome this limitation, appealing approaches based on dopant-free, self-doping Ti^3+^ species in TiO_2_ have recently been developed. No foreign elements are introduced in Ti^3+^ self-doped TiO_2_, which increases convenience. Moreover, numerous oxygen vacancies are beneficial for amplifying absorption in the visible region by reducing the bandgap and increasing electron mobility. Ti^3+^ self-doped TiO_2_ is easy to prepare compared with common doped forms of TiO_2_^11,14,20–23^. Previous research has focused on modifying and preparing TiO_2_ with carbon nanotubes (CNTs) as composites to reduce recombination of photoexcited e^−^/h^+^ pairs. CNTs are composed of *sp*^2^ hybrid carbon atoms, which have a large surface area, exceptional electrical properties, and high charge mobility. TiO_2_ nanoparticles (NPs) coupled with CNTs exhibit excellent photocatalytic activity. Carbon functions as an electron trapper, enhancing the conductivity of TiO_2_, minimizing charge recombination, and promoting electron–hole separation. The coupling of TiO_2_ with CNTs can increase quantum efficiency because it (1) results in the formation of a heterojunction that hinders e^−^/h^+^ pair recombination; (2) enables VL absorption by forming Ti–C or Ti–O–C defect sites that act as an impurity; and (3) provides more e^−^ to the conduction band of TiO_2_ by creating e^−^/h^+^ pairs under incident light^13,14,17,21–24^.

Here, we report a facile two-step chemical precipitation and calcination method for the selective preparation of Ti^3+^/TiO_2_/CNT and Ti^3+^/TiO_2_/C (C in Ti^3+^/TiO_2_/C is a carbon layer) nanocomposites. All of the reagent amounts (except CNTs) and calcination conditions were the same in the preparation methods. The nanocomposites were characterized and the photocatalytic efficiency of all the nanocomposites was examined through MB dye decomposition under VL, revealing substantial photocatalytic efficiency. The effectiveness of TTOC-2 (Ti^3+^/TiO_2_/C) composite toward the degradation of RhB, CR, MO, and phenol was subsequently evaluated. In the reusability test, no notable activity deterioration was observed after five consecutive runs. The novelty of the present work involves finding a new route for the selective preparation of Ti^3+^/TiO_2_/CNT and Ti^3+^/TiO_2_/C nanocomposites. The process is developed here for the first time, and no previous reports have used identical techniques. Moreover, all the nanocomposites show excellent photocatalytic activity toward organic pollutant degradation and the present method overcomes the shortcomings of TiO_2_ as a photocatalyst under visible light.

## Results and discussion

### Characterization of the samples

**Figure 1:**
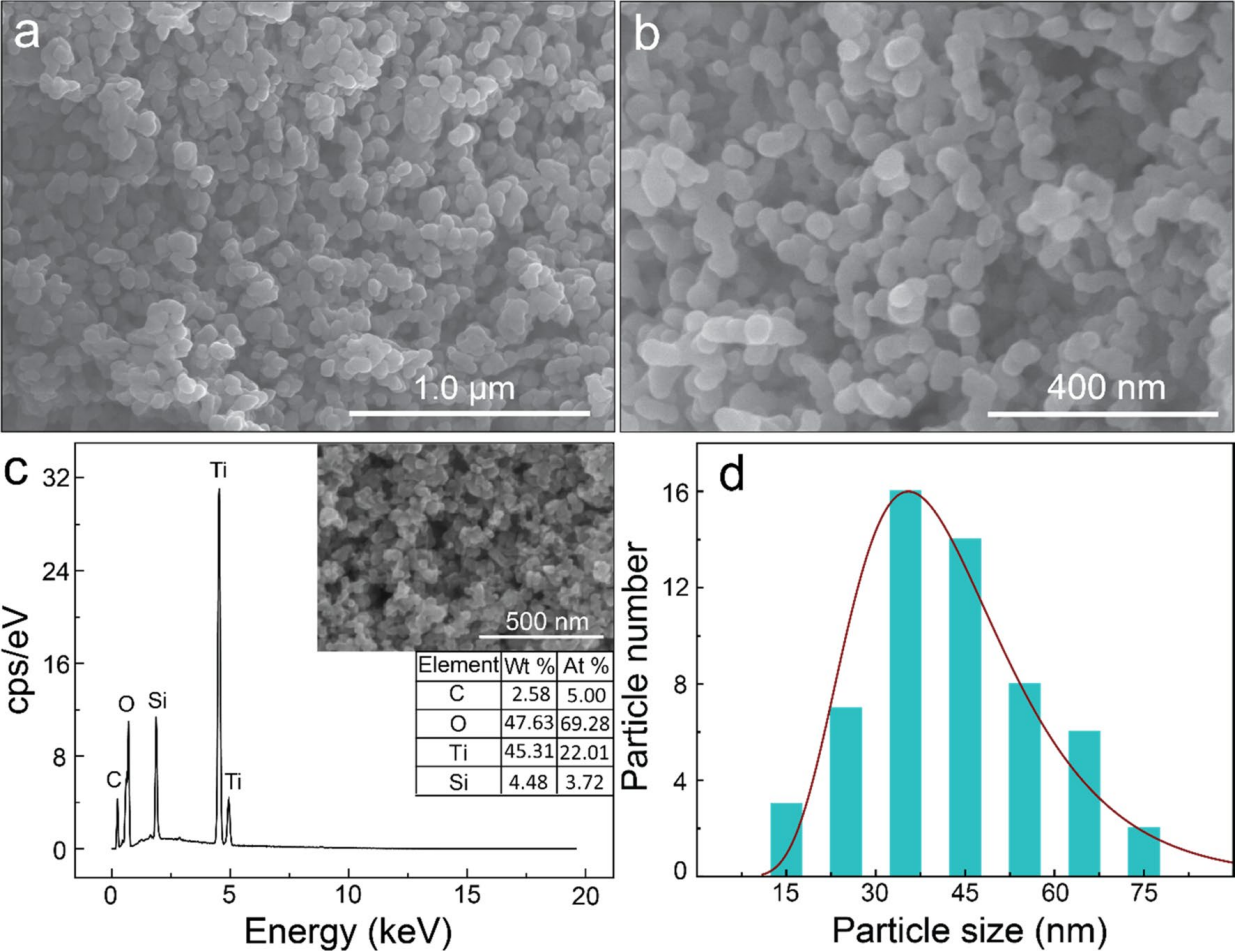
(**a**) Low-resolution and (**b**) high-resolution FE-SEM images, (**c**) EDS curves (the inset shows the corresponding area evaluated by EDS, along with the tabulated data), and (**d**) size distribution of the TTOC-2 nanocomposite.

To demonstrate the surface topography, field-emission scanning electron microscopy (FE-SEM) images of the TTOC-2 nanocomposites at different magnification are presented in Fig. 1a,b. The FE-SEM micrographs show that the as-synthesized compound is composed of irregular spherical-shaped NPs with diameters ranging from ~ 15 to ~ 75 nm and a mean diameter of 43 nm (Fig. 1d). The chemical composition of the TTOC-2 nanocomposite was confirmed from its energy-dispersive X-ray spectrum (EDS) (Fig. 1c). The inset FE-SEM image in Fig. 1c was used for EDS analysis, and the elemental composition is presented in the table (inset). The EDS spectrum affirms the presence of carbon species with Ti and O elements. The precursor CNT was not observed in the FE-SEM analysis; however, the EDS studies confirm the presence of carbon in TTOC-2, indicating breakdown of the CNTs. EDS of the TTOC-2 sample was conducted on the silicon wafer instead of carbon tape for more accurate analysis. A signal of Si at ~ 1.83 keV was observed from the silicon wafer.

**Figure 2:**
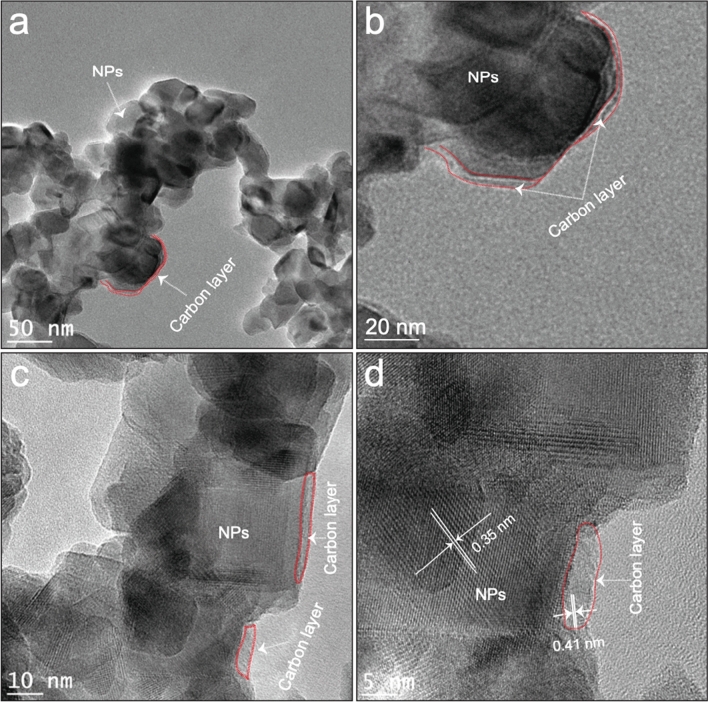
(**a**), (**b**), and (**c**) TEM images of TTOC-2 at different magnifications; (**d**) high-resolution TEM image of TTOC-2 nanocomposites.

Transmission electron microscopy (TEM) analysis was conducted to further analyze the state of the CNTs in the TTOC-2 sample; images at three different magnifications are shown in Fig. 2a–c. The results confirmed the nanostructure of the catalyst and explored the absence of CNTs. The appearance of the nanocomposites suggests the presence of layer around the particles. The crystalline nature of nanoparticles and the amorphous nature of the layer were discernably observed. Both FE-SEM and TEM results indicate the formation of carbon layer from CNTs precursor used in the preparation. The different forms of carbon can be found through TEM imaging. The formation of a thin carbon layer (marked with a red dashed line) was clearly observed in Fig. 2. High-resolution TEM (HR-TEM) analysis was subsequently used to observe the crystal lattice (Fig. 2d), which confirms the existence of two different conjoint planes. The estimated *d*-spacing matches the interplanar spacing of graphitic carbon^1^, and the *d*-spacing (0.35 nm) is assigned to the (101) facet of anatase TiO_2_^25^. These findings are significantly different from that of TTOC-1 and TTOC-3 compounds.

FE-SEM images of the TTOC-1 nanocomposite are shown in Supplementary Figures S2a and S2b. The densely packed NPs were observed to uniformly coat the CNT surface. FE-SEM images of TTOC-3 are presented in Supplementary Figures S2c and S2d. CNTs are clearly observed to homogeneously decorate the nanocomposite surface. The morphology of the TTOC-1 nanocomposite is similar to that of the TTOC-3 nanocomposite even though the amount of CNTs in the composites differs. The EDS spectra of TTOC-1 and TTOC-3 are shown in Supplementary Figures S3a and S3b, respectively. The respective FE-SEM images used for the EDS analysis and the tables detailing the elemental compositions are shown in the insets of the figures. Both spectra indicate the presence of O, Ti, and C. A signal of Si was found due to silicon wafer used in the analysis process.

The TTOC-1 and TTOC-2 nanocomposites were studied by TEM. Supplementary Figure S4a shows TEM images of TTOC-1. The spherical NPs with a mean diameter of 51 nm are superimposed on the CNTs. In the HR-TEM image of (Supplementary Figure S4b), the lattice *d*-spacing of 0.35 nm is assigned to the (101) plane of TiO_2_ NPs and that of 0.41 nm is ascribed to the graphitic carbon of CNTs. A TEM image of TTOC-3 is shown in Supplementary Figure S4c; the growth of TiO_2_ NPs with a mean diameter of 47 nm is clearly observed. The same lattice fringe spacings observed for TTOC-1, 0.35 nm and 0.41 nm, are also observed for TTOC-3 and are attributed to the TiO_2_ NPs and the CNTs, respectively (HR-TEM image, Supplementary Figure S4d).

**Figure 3:**
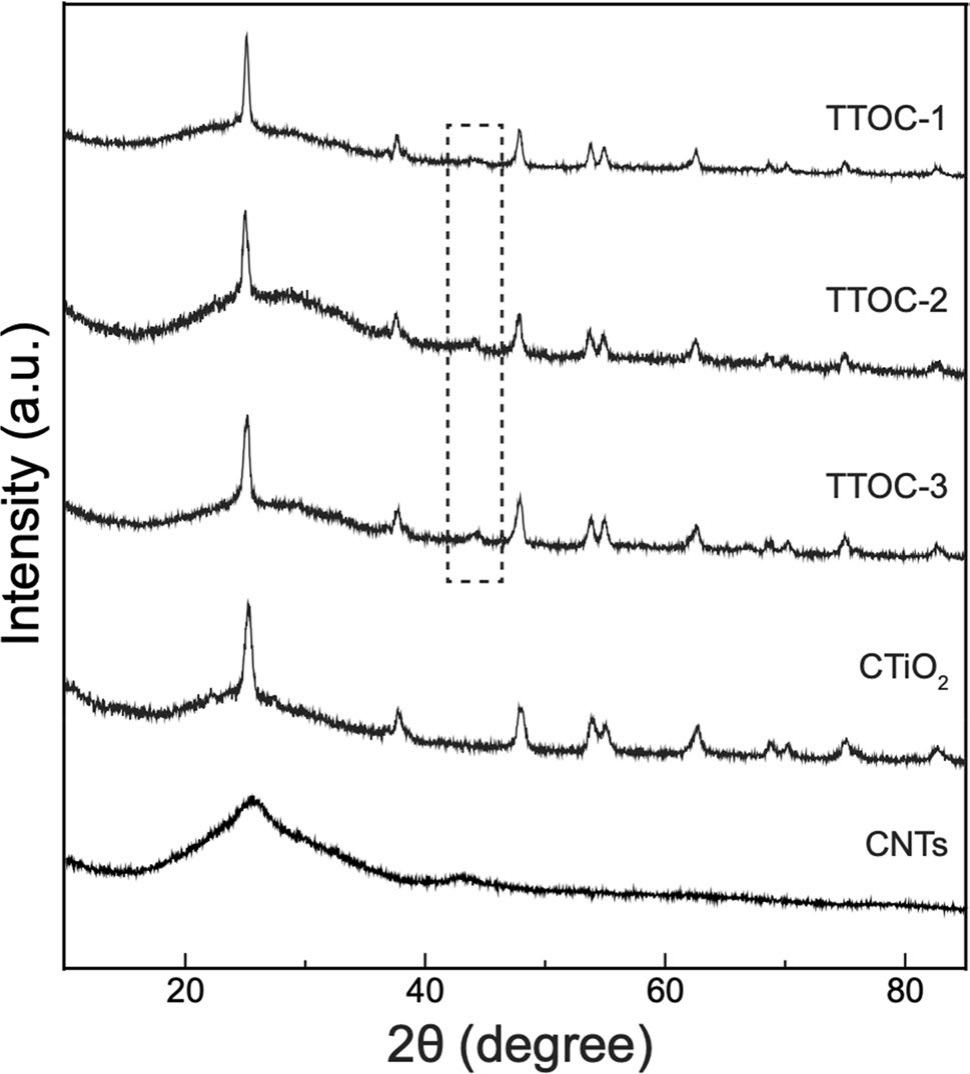
XRD patterns of the as-synthesized nanocomposites (TTOC-1, TTOC-2, and TTOC-3), commercial TiO_2_ (CTiO_2_), and pristine CNTs.

The structure and phase purity of the photocatalysts was studied by X-ray diffraction (XRD). The XRD plots of the nanocomposites are displayed in Fig. 3. For comparison, the XRD patterns of commercial anatase TiO_2_ (CTiO_2_) and pristine CNTs are also presented. The two characteristic peaks of the CNTs, centered at 2*θ* angles of 25.28° and 44.27°, are indexed to their (002) and (100) crystal planes, respectively^26^. The first broad peak corresponds to interlayer stacking, and the second, weaker peak is attributed to the interplanar stacking of aromatic systems. The pattern of the commercial TiO_2_ shows diffraction peaks at 25.28°, 37.56°, 47.77°, 53.72°, 54.73°, 62.51°, 68.65°, 70.07°, 74.79°, and 82.53°, which can be assigned to the (101), (004), (200), (105), (211), (204), (116), (220), (215), and (224) planes, respectively. They are all signature peaks of anatase tetragonal TiO_2_ (JCPDS card No. 96–500-0224) with space group *I*_41_/*amd*. The diffraction patterns of nanocomposites TTOC-1 and TTOC-3 are well indexed to a combination of anatase TiO_2_ and CNTs. The presence of a carbon species (2*θ* = 25.28° and 44.27°) and anatase TiO_2_ was also observed in the TTOC-2 nanocomposite. The characteristic peaks of rutile TiO_2_ were absent. The most common forms of TiO_2_ are the rutile and anatase polymorphs. According to the literature, anatase exhibits greater photocatalytic activity than rutile. Notably, the synthesized nanocomposite contains pure anatase TiO_2_. The identification of the most intense diffraction peak of carbon (2*θ* = 25.28°) in the patterns of the nanocomposites was difficult because of overlap with the most intense diffraction peak of TiO_2_. However, the asymmetry and broadening of the 25.28° peak of TiO_2_ in the nanocomposites reveal the effect of CNT/carbon species on the diffraction pattern of TiO_2_. The peaks in the black-dotted rectangle clearly indicate the presence of carbon species (2*θ* = 44.27°; (100) plane) in all of the prepared nanocomposites.

**Figure 4:**
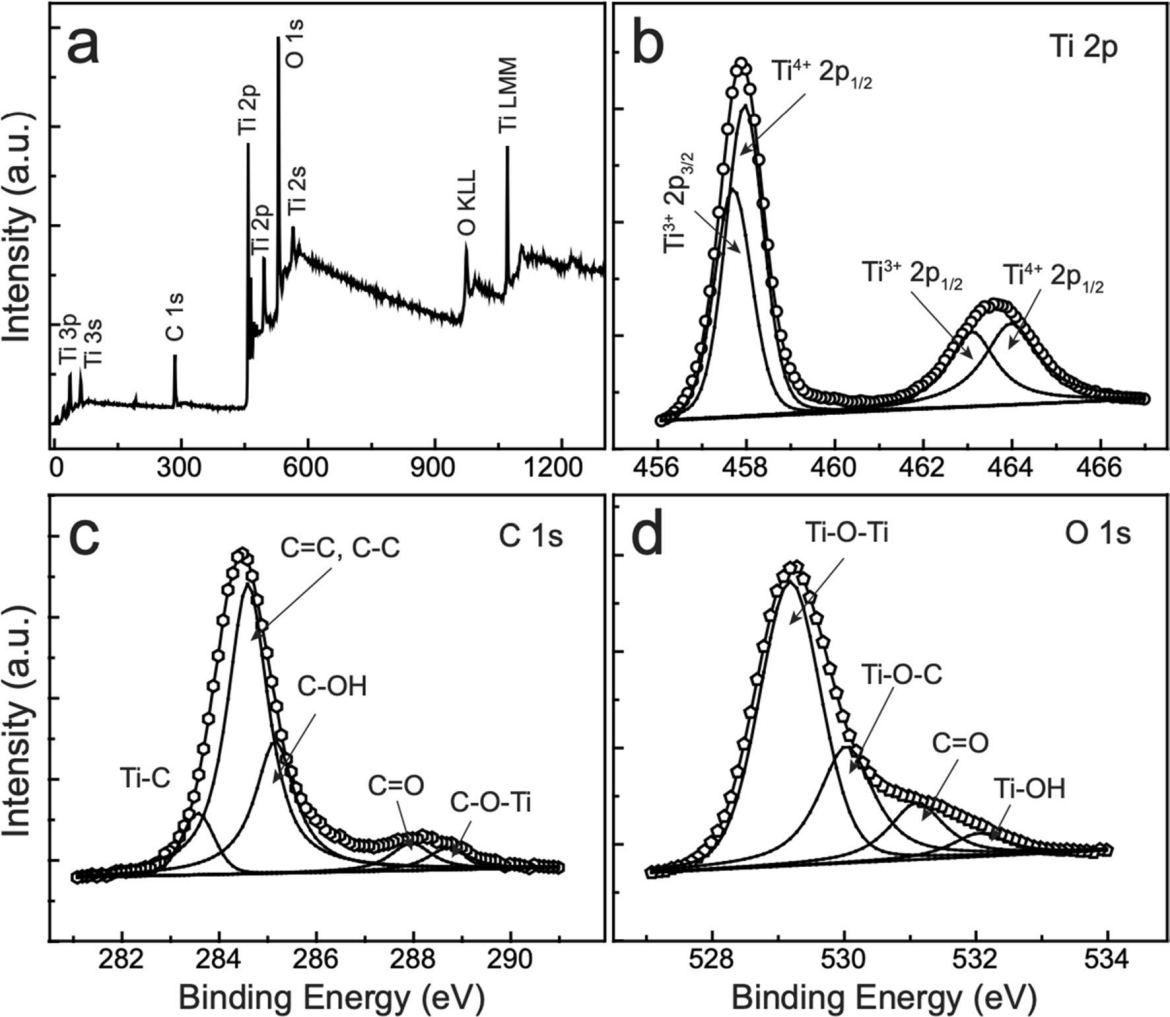
(**a**) XPS survey pattern and (**b**) Ti-2*p*, (c) C-1*s*, and (**d**) O-1*s* core-level XPS spectra of the TTOC-2 nanocomposite.

The surface composition of the nanocomposites was evaluated by X-ray photoelectron spectroscopy (XPS); the results are shown in Fig. 4, Supplementary Figures S5, and S6 for TTOC-2, TTOC-1, and TTOC-3, respectively. The survey spectrum affirms the presence of Ti, C, and O in the TTOC-2 (Fig. 4a), TTOC-1 (Supplementary Figure S5a), and TTOC-3 (Supplementary Figure S6a) nanocomposites. The Ti-2*p* XPS spectra of all of the nanocomposites (Fig. 4b, Supplementary Figures S5b and S6b) show two peaks at 457 and 463 eV. However, the Ti-2*p* XPS signals of Ti^4+^ should be located at ~ 459.0 and ~ 464.5 eV. This shift in the binding energy of Ti^4+^ to lower energies suggests the presence of Ti^3+^ dopant. Deconvolution of the Ti-2*p* peaks reveals the presence of both Ti^4+^ and Ti^3+^ in the samples. The signals at 463.08 and 457.73 eV are assigned to Ti-2*p*_1/2_ and Ti-2*p*_3/2_ of Ti^3+^, whereas the modes at 464.02 and 459.09 eV are attributed to the Ti-2*p*_1/2_ and Ti-2*p*_3/2_ of Ti^4+^, respectively^11^. A Gaussian fitting of the Ti-2*p* peaks was used to estimate the relative Ti^3+^ content of the nanocomposites quantitatively. Small shoulders of the peaks associated with Ti^3+^ species compared with the peaks associated with Ti^4+^ species are noticeable in the spectra of all of the nanocomposites. The calculated Ti^4+^:Ti^3+^ ratio for TTOC-1, TTOC-2, and TTOC-3 is 1:0.55, 1:0.74, and 1:0.57, respectively. These results indicate that Ti^3+^ is more prevalent in TTOC-2 than in TTOC-1 and TTOC-3. The large Ti^3+^ content in TTOC-2 confirms the high stability of the produced Ti^3+^ ions. The stability of Ti^3+^ increases because of the presence of a carbon layer around the Ti^3+^/TiO_2_ particles in the TTOC-2 nanocomposite. Ti^3+^ species are present in the TiO_2_ as a lattice Ti^3+^ and surface Ti^3+^. The stability of surface Ti^3+^ is less stable than a lattice Ti^3+^ because of easy oxidation in contact with air. The prospect of Ti^3+^ oxidation reduces by the formation of the carbon layer. The carbon layer acts as a shield/barrier for surface Ti^3+^ species of TiO_2_, which reduces the Ti^3+^ conversion to other forms and helps to maintain the content of Ti^3+^ species. In TTOC-1 and TTOC-3, Ti^3+^ ions are located on or near the surface of TiO_2_, enabling their easy oxidation to Ti^4+^ and thereby reducing the peak area of Ti^3+^ ions. The C-1*s* and O-1*s* fitting XPS spectra of all the nanocomposites discussed in the Supplementary section 2 (Supplementary Figures S5 and S6).

The functional groups investigation of the nanocomposites by Fourier transform infrared (FTIR) spectroscopy confirmed the presence of carbon species and TiO_2_ in all the prepared nanocomposites; this is discussed in the Supplementary Section 3 (Supplementary Figure S7).

**Figure 5:**
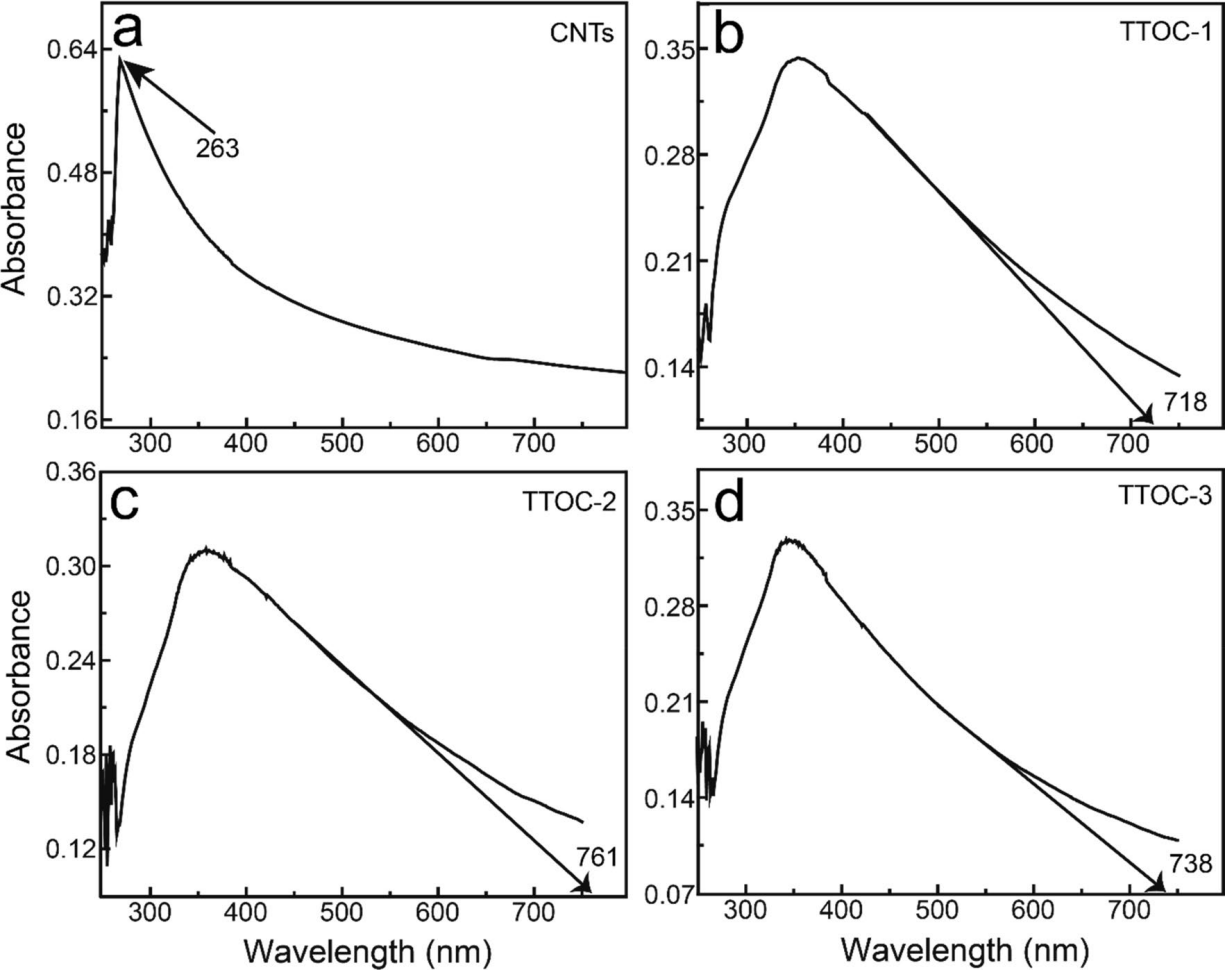
UV–vis spectra of (**a**) pristine CNTs and (**b**) TTOC-1, (**c**) TTOC-2, and (**d**) TTOC-3 nanocomposites.

UV–vis absorbance was employed to investigate the optical features of the nanocomposites. Figure 5a–d shows the UV–vis absorbance of pristine CNT, TTOC-1, TTOC-2, and TTOC-3, respectively. The pristine CNT shows a characteristic absorption peak at 263 nm. The absorption edge of TTOC-1, TTOC-2, and TTOC-3 nanocomposites was observed at 718, 761, and 738 nm, respectively. The absorption-edge wavelength (*λ*_g_) was calculated from the intercept between the abscissa coordinate and the tangent of the absorption curve. The absorbance of all the nanocomposites shows almost full-range coverage of VL wavelengths. Among them, the TTOC-2 nanocomposite exhibits the highest *λ*_g_. The extended VL absorption range might be dependent on the Ti^3+^ oxygen/vacancy states on the TiO_2_ surface. The carbon layer improves the stability of the Ti^3+^ in the TTOC-2 composite compared with that in the TTOC-1 and TTOC-3 composites.

The bandgaps of the nanocomposites and pristine CNT were analyzed from the following Tauc equation, (*αhv*)^n^ = A(*hv*-*E*_g_); where *E*_g_, A, *hv*, and *α* are the bandgap energy, constant, incident photon energy, and absorption coefficient, respectively. The value of n depends on the transition feature of electrons (indirect transition: n = 1/2; direct transition: n = 2). *E*_g_ was calculated from plots (*αhʋ*)^2^ vs. *E*, where the intercept to the *E* axis denotes *E*_g_ where (*αhʋ*)^2^ = 0. Figures S8a–S8d show the Tauc plots of pristine CNT, TTOC-1, TTOC-2, and TTOC-3. The calculated bandgaps of the CNTs and the TTOC-1, TTOC-2, and TTOC-3 nanocomposites are 3.47, 2.08, 1.93, and 2.04 eV, respectively. To ensure the starting at zero levels, the plots of transformed Kubelka–Munk function vs *E* were also demonstrated. As shown in Supplementary Figure S9(a-c), the *E*_g_ values of all the nanocomposites matched well with those obtained from absorption data (± 0.07). The bandgap of the nanocomposites is dramatically lowered by the introduction of carbonaceous species and Ti^3+^ ions. This smaller bandgap makes the nanocomposites applicable in the visible range, which is one of the criteria for a good photocatalyst. In the case of a small bandgap, low-energy light is sufficient to excite valence-band (VB) electrons into the conduction band (CB). The lowest bandgap of TTOC-2 among the nanocomposites is attributed to the formation of a carbon layer.

The photoluminescence (PL) analysis was used to investigate immigration, transfer or the fate of (*e*–*h*) pairs, and efficiency of charge carrier trapping; this is discussed in Supplementary Section 4 (Supplementary Figure S10).

The surface area and pore size were assessed by N_2_ absorption–desorption isotherm analysis. The Brunauer–Emmett–Teller (BET) surface area and pore size of the pristine CNTs are 255.39 m^2^/g and 79.79 Å, respectively. The high surface area of the CNTs decreased in all of the TTOC samples, suggesting the formation of composites.

The BET surface area of the TTOC-1, TTOC-2, and TTOC-3 nanocomposites was 23.53, 29.95, and 24.86 m^2^/g, respectively. The reason for the relatively greater surface area of TTOC-2 compared with those of TTOC-1 and TTOC-2 is the formation of small Ti^3+^/TiO_2_ NPs. The carbon layer is the driving force for the formation of small-sized NPs. The average pore size of the TTOC-1, TTOC-2, and TTOC-3 nanocomposites was 264.00, 401.23, and 394.79 Å, respectively. The pore size depends on the Ti^3+^ ions; that is, a high content of Ti^3+^ indicates a large pore content. The obtained results are consistent with this relationship. Large pores of a catalyst promote the adsorption of organic molecules on its surface during photodegradation, thus increasing its photocatalytic activity^27^. The surface area and pore size values are presented in Table 1. The adsorption–desorption isotherms of the pristine CNTs and the TTOC-1, TTOC-2, and TTOC-3 nanocomposites showed in Supplementary Figures S11(a–d), respectively and discussed in Supplementary Section 5.

**Table 1:** BET surface area and pore size of the pristine CNTs and the nanocomposites.

Sample	Surface area (m^2^/g)	Pore size (Å)
CNTs	255.3898	79.7895
TTOC-1	23.5293	264.8500
TTOC-2	29.9555	401.2321
TTOC-3	24.8571	395.7971

### Visible-light-driven photocatalytic performance of the nanocomposites

**Figure 6:**
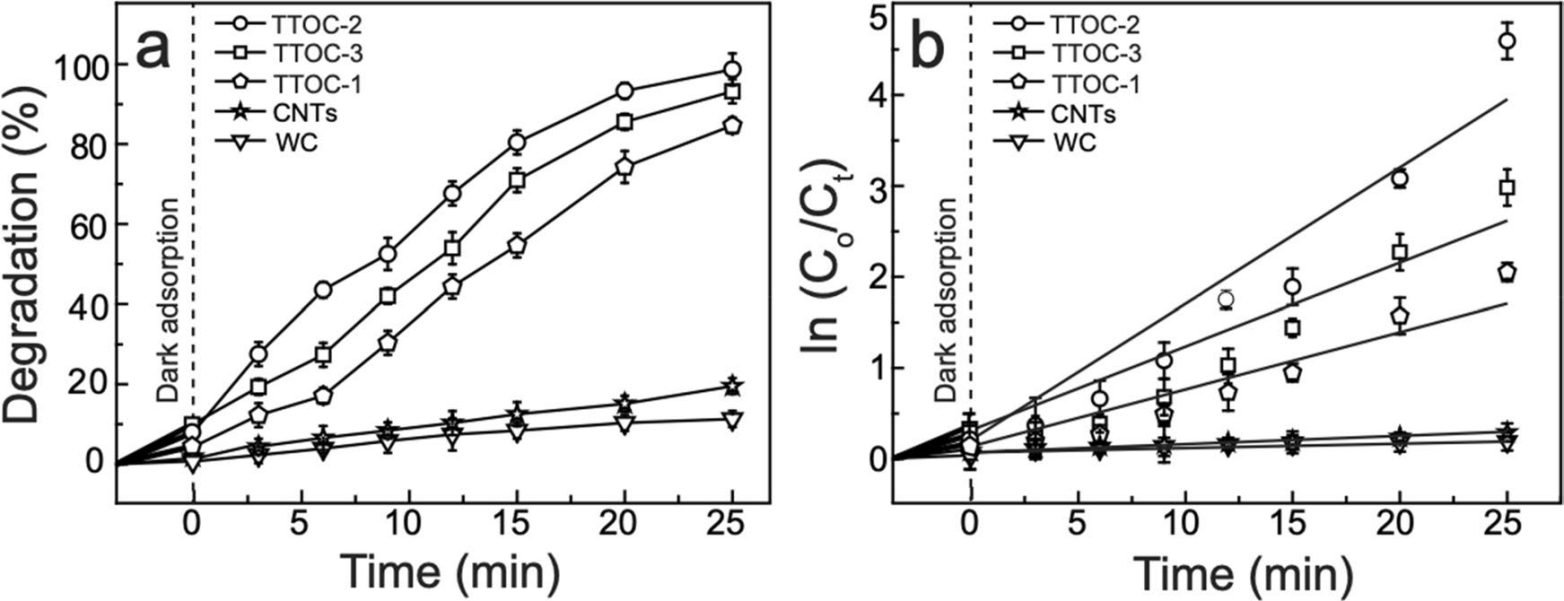
Photocatalytic performance of the nanocomposites (TTOC-1, TTOC-2, and TTOC-3), pristine CNTs, and WC under visible light: (**a**) variation of the degradation percentage over the illumination time and (**b**) the first-order photochemical reactions kinetics. Error bars in (**a**) and (**b**) represent standard deviations for three runs.

The photocatalytic performance of the TTOC-1, TTOC-2, and TTOC-3 nanocomposites and pristine CNTs was evaluated on the basis of MB degradation under irradiation by a Xe lamp. MB was chosen because it is persistent and widely used in various industries. For comparison, a blank reaction without a catalyst (WC) was also conducted. Figure 6a shows the degradation percentage over the illumination time; the first-order kinetics of the reaction are represented in Fig. 6b. The MB degradation percentage was approximately 83%, 98%, and 93% for the TTOC-1, TTOC-2, and TTOC-3 nanocomposites, respectively, after 25 min of VL irradiation. When the reaction time was prolonged to 35 min using TTOC-2, the characteristics peak of MB at λ_max_ = 664 completely disappeared; indicates the ~ 100% removal of MB. The self-deterioration of MB was trivial under irradiation of VL. In addition, the pristine CNTs showed no noticeable photocatalytic activity. The change in MB concentration under dark conditions was also measured at regular time intervals. During the adsorption–desorption period, the reduction of the MB concentration after 25 min was negligible. The reaction rate of the MB decomposition on the pristine CNTs and the TTOC-1, TTOC-2, and TTOC-3 nanocomposites was 0.0083, 0.07, 0.15, and 0.10 min^−1^, which is 166%, 1400%, 3000%, and 2000% greater, respectively, than the rate of the WC reaction (0.005 min^−1^). As shown in Supplementary Table S1, the correlation coefficient value (R^2^) of Fig. 6b signifies the smooth photocatalytic reaction. All of the prepared nanocomposites showed greater photocatalytic activity because of the coexistence of Ti^3+^ and carbon species along with TiO_2_. The bandgap of Ti^3+^-TiO_2_ differs from that of pure TiO_2_ and can utilize a wide wavelength range of VL radiation for exciting the VB electrons. In addition, the carbon species increased the adsorption of pollutants and reduced the e^−^/h^+^ recombination rate, thus enhancing the photodegradation efficiency. Because of its greater CNT content, TTOC-3 exhibited greater photocatalytic activity than TTOC-1. However, the photocatalytic performance of TTOC-2 was better than that of TTOC-1 and TTOC-3. In TTOC-2, the Ti^3+^ stability is improved by the presence of a carbon layer around the Ti^3+^-TiO_2_ NPs. The carbon layer also substantially decreases the particle size and simultaneously increases the specific surface area. A high specific surface area enhances the photodegradation efficiency because the reaction occurs at the surface. Discoloration images of an MB solution in the presence of the TTOC-1, TTOC-2, and TTOC-3 nanocomposites are presented in Supplementary Figures S12a, S12b, and S12c, respectively. The results confirm the excellent changes in MB concentration within short periods.

In addition, the degradation activity of TTOC-2 toward CR, RhB, phenol, and MO was investigated under similar experimental conditions. The photodegradation ratio (*C*_*t*_/*C*_0_) over the illumination time was plotted in Supplementary Figure S13a. Within 25 min, only 28% of the MO was degraded, whereas 71% of the RhB was degraded. The non-photosensitizing CR dye and colorless organic pollutant phenol show 91% and 69% photodegradation. The linear relation of ln(*C*_0_/*C*_*t*_) vs illumination time (Supplementary Figure S13b) confirms the first-order reaction kinetics. Also, the high coefficient value (R^2^) (Table S1) of Supplementary Figure S13b confirms the smooth flow of the reaction. The rate constants of the CR, RhB, phenol, and MO degradation reactions were 0.0808, 0.0452, 0.0412, and 0.0147 min^−1^, which are 1616%, 904%, 824%, and 307% higher than the rate constants of the corresponding reactions without a catalyst. The low degradation rate of MO is due to the presence of an azo bond, which is difficult to rupture^28,29^.

### Effect of pH and point of zero charge (PZC) on photocatalytic degradation

The determination of the pH at the point-of-zero-charge (pH_PZC_) and an evaluation of the effect of pH on photodegradation are critical. The pH of the mixture affects the solubility of dyes and the surface chemistry of the adsorbent. The pH_PZC_ demonstrates a sample's surface charge. The drift method was used for pH_PZC_ calculation in the pH range between 2 and 12. HCl and NaOH were employed to control the pH of the solution. Supplementary Figure S14 shows a graph of (pH_i_ − pH_f_) vs pH_i_, where pH_i_ and pH_f_ and the initial and final pH, respectively. The measured pH_PZC_ (where the final pH is equal to the initial value) was 9.31, 9.92, and 9.63 for TTOC-1, TTOC-2, and TTOC-3, respectively. These results imply that the nanocomposites are cationic at pH levels below the pH_PZC_ and anionic at pH levels greater than the pH_PZC_. To verify this speculation, the effect of pH on the degradation of MB by TTOC-2 was evaluated in the range 2 ≤ pH ≤ 12 under similar experimental conditions; the results are presented in Supplementary Figure S15. Superior photodegradation was observed at pH 12, whereas the worst performance was obtained at pH 2 (Supplementary Figure S15a). The photodegradation ratio clearly increases with increasing pH because MB is cationic at pH values greater than 5.8 (pKa = 5.8). In a basic medium, the electrostatic attraction between the cationic MB and the catalyst's negative surface increases. The opposite effect is observed in an acidic medium, and the photocatalytic efficiency is decreased. The results of kinetics studies of the effect of pH on MB degradation are shown in Supplementary Figure S15b. The linear relation between ln(C_0_/C_*t*_) and irradiation time confirms first-order kinetics. The rate constant of the reaction at pH 2, 5, 9, and 12 was 0.0163, 0.0314, 0.0754, and 0.1454 min^−1^, respectively. The rate constant of the reaction at pH 12 was 892% greater than that of the reaction at pH 2. The superior photodegradation of the as-synthesized nanocomposites at high pH levels also confirms the presence of negative surface groups when the nanocomposites are in a basic medium. Discoloration images of MB solution at pH 2 (after 25) and pH 12 (after 20 min) are presented in Supplementary Figures S12d and S12e, respectively.

In addition to the efficiency of a photocatalyst, its stability and reusability are also essential parameters for evaluating its performance. Reusability experiments were performed using recovered nanocomposites. The degradation ratio over five consecutive cycles is presented in Supplementary Figure S16a. No significant changes were observed throughout the runs. The degradation efficiencies after five cycles were 81.0%, 96.0%, and 90.2% for TTOC-1, TTOC-2, and TTOC-3, respectively. The XRD patterns of the three nanocomposites were collected (Supplementary Figure S16b) after five consecutive runs and showed no obvious differences from the patterns of the fresh samples. The XRD crystal planes of the samples before reaction and after the reaction are consistent. The low-intensity characteristic peaks of the (100) plane of carbon species (2*θ* = 44.27°) are also observed. These results indicate excellent reusability and stability of the nanocomposites.

The photodegradation activity of the as-synthesized nanocomposites is compared with that of various reported catalysts in Supplementary Table S2. This comparison demonstrates that our composites show substantial photocatalytic activity under VL.

### LC–MS analysis

The intermediate products in the MB degradation were examined through liquid chromatography–mass spectrometry (LC–MS) experiments. The mass spectra of an aqueous MB solution after photocatalytic reaction (25 min) with the TTOC-2 nanocomposite are presented in Supplementary Figure S17. The characteristic *m*/*z* 284 of MB was hardly observed in the mass spectrum collected after photodegradation. The high-energy electrons and OH^•^ are responsible for the deterioration of MB. Structures of the MB degradation intermediate products were proposed on the basis of their *m*/*z* ratios. The direct hydroxylation of MB is dominant, and products at *m*/*z* ratios of 338 and 384, which are greater than that of MB, were obtained. The other hydroxylated and demethylated products were detected at *m*/*z* ratios of 325, 265, and 255. The degradation products at *m*/*z* ratios of 295 and 312 were obtained through rupture of the C–$S^{ + } {-}$ C bonds by OH^•^ radicals (oxidation reaction). The successful rupture of the MB molecule was supported by the presence of smaller *m*/*z* peaks at 110, 168, 177, and 217. The presence of the peaks at *m*/*z* ratios of 145 and 102 indicates the complete breaking up of aromatic rings in MB. The smaller fragmented products lead to total degradation of the MB molecules into nontoxic acids (e.g., acetic acid and oxalic acid) or to mineralized inorganic substances (e.g., CO_2_, H_2_O, SO_4_^2−^, and NO_3_^−^). A possible pathway of MB degradation based on the intermediate products obtained in the mass spectra is displayed in Supplementary Figure S18. The results thus confirm the effectiveness of the as-prepared nanocomposites as photocatalysts for MB dye degradation.

**Figure 7:**
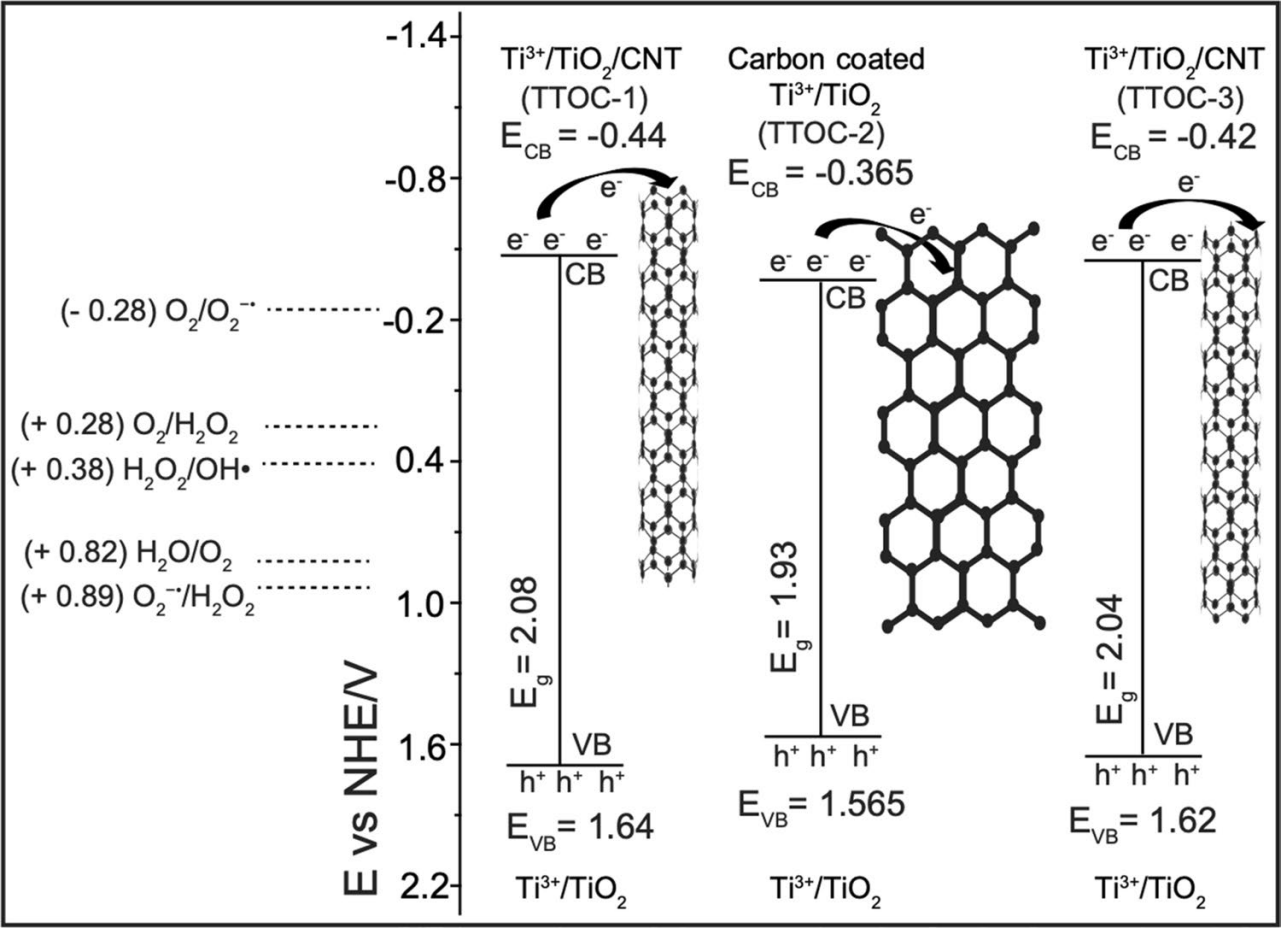
Schematic of the redox couples and energy levels of the nanocomposites.

CB and VB edge potential was estimated to illustrate the photocatalytic mechanism of as-synthesized photocatalyst. Proposed mechanism path of photocatalytic performance was discussed in Supplementary Section 6. The schematic reaction mechanism with redox couples and energy band positions is shown in Fig. 7.

Further, scavenger studies were carried out to elucidate the involvement of active species in the photocatalytic reaction mechanism and discussed in Supplementary Section 7 (Supplementary Figure S19).

## Conclusions

In summary, we fabricated Ti^3+^/TiO_2_/CNT and Ti^3+^/TiO_2_/C nanocomposites using a straightforward precipitation and calcination process. The amount of CNTs was varied, whereas the loading amount of other precursors was kept constant. The nanocomposites' *E*_g_ was remarkably low, and their absorbance covers the entire visible-light wavelength range. The average size of the Ti^3+^/TiO_2_ particle in all composites was less than 100 nm, resulting in a high specific surface area. The photocatalytic efficiency of the nanocomposites was tested for the degradation of a MB solution under VL. All of the nanocomposites showed high photocatalytic efficiency within 25 min: 83%, 98%, and 93% for the TTOC-1, TTOC-2, and TTOC-3 nanocomposites, respectively. The photocatalytic efficiency enhancement was attributed to the introduction of Ti^3+^ and carbon species onto TiO_2_. Among the nanocomposites, TTOC-2 exhibited the highest activity because of its large Ti^3+^ content as a result of the formation of a carbon shell. Similar experiments with TTOC-2 for the degradation of CR, RhB, phenol, and MO were performed, resulting in ~ 91%, ~ 71%, ~ 69%, and ~ 28% degradation, respectively. The PZC of the nanocomposites revealed a negative nature of their surface at high pH. The effect of pH on MB degradation using TTOC-2 was also demonstrated. With increasing pH value, the photocatalytic activity increased. Moreover, after five consecutive cycles, no apparent loss of photocatalytic activity was observed and the XRD patterns showed no structural changes, indicating good cycling stability. Therefore, the proposed nanocomposites are suitable for practical application in wastewater treatment because of their high stability and high photocatalytic efficiency. In addition, the selective preparation techniques for the two different nanocomposites might be useful in the preparation of future photocatalysts.

## Experimental methods

### Selective preparation of Ti^3+^/TiO_2_/CNT and Ti^3+^/TiO_2_/C nanocomposite

Titanium(IV) isopropoxide (TTIP), sodium borohydride (NaBH_4_), RhB, CR, phenol, benzoquinone (BQ), isopropyl alcohol (IPA), potassium iodide (KI), and ethanol were sourced from Sigma-Aldrich (USA). The single-walled carbon nanotubes (CNTs, outer diameter: 1–2 nm, length: 5–30 µm) were purchased from US Research 307 Nanomaterials (Houston, USA). MB was obtained from Alfa Aesar (UK). The nanocomposites were prepared using a convenient and facile two-step precipitation and calcination process with TTIP and CNT as precursors. In the first step, 5 mg of CNTs was dispersed in 25 mL of ethanol using a sonication bath. Five milliliters of TTIP was then applied to the dispersed CNTs under continuous stirring. A 100 mL aqueous mixture of 0.10 g NaBH_4_ was then slowly poured into the solution. The mixture was covered with Al foil and then vigorously stirred for 3 h at 600 rpm on a magnetic stirrer. The precipitate was rinsed with distilled water and dried overnight at 60 °C. Subsequently, in the second step, the precipitate was calcined at 550 °C for 6 h with a ramp rate of 7.5 °C/min to obtain a stable composite. During calcination, the sample was placed in a lid-protected crucible, which further protected in the stainless-steel chamber. The chamber was closed by an airtight UHV seal of clean, highly purified copper gaskets (oxygen-free high conductivity). At the end, the locked chamber was positioned in the furnace. The thus-obtained nanocomposite was labeled as TTOC-1. Three nanocomposites were prepared with different mass loadings of CNTs; the other reaction conditions were unchanged. During dispersion, the volume of ethanol was increased proportionally with increasing amount of CNTs. The obtained products with CNT loadings of 10, 15, and 20 mg were denoted as TTOC-1, TTOC-2, and TTOC-3, respectively. A reaction mechanism is proposed for the preparation of the two different nanocomposites (Supplementary Figure S1). Hydrolysis of TTIP (strong Lewis acid) generally produces TiO_2_-NPs. The highly electronegative isopropoxide (− OCH_3_)_3_ groups undergo protonation reaction, and on the other hand, Ti − OH bond formation occurs. The functional group of Ti–OH provides stronger binding with CNTs. Subsequently, TiO_2_-NPs were produced through hydrolysis and attached to CNTs through chemical bonding. Here, NaBH_4_ was used to reduce Ti^4+^ ions on the surface of TiO_2_ to Ti^3+^ ions. The precipitation reaction produced three Ti^3+^/TiO_2_/CNT composites with different CNT loadings. However, after calcination, the CNTs ruptured and produced a carbon layer around the Ti^3+^/TiO_2_-NPs only in the TTOC-2 nanocomposite. The aforementioned analysis indicates that, among the nanocomposites, TTOC-2 exhibits the strongest interaction between the Ti^3+^ doped TiO_2_ and the CNTs. The characteristics of the titanium oxide and CNTs composites vary depending on individual reaction procedures, synthesis conditions, and the mass ratio of the precursor. The reproducibility of the method was checked by repeating the process several times; in each case, the results were identical. Characterization methods are described in Supplementary Section 1 in detail.

### Investigation of photocatalytic activity

The photocatalytic efficiency of the samples was tested via the degradation of MB, RhB, CR, MO, and phenol using a 300 W Xe lamp as a solar-light simulator. A 100 mL aqueous solution containing 10 mg of the pollutant was mixed with 0.05 g of catalyst under ultrasonication for 1 h. The mixture was then placed in the dark for 1 h to ensure that adsorption/desorption equilibrium was achieved. The mixture was irradiated under a Xe lamp for 25 min. A 400 nm UV cutoff filter was used to prevent irradiation with UV light. A fixed amount of solution was collected at regular time intervals, and the absorbance of the solution was monitored using a UV–vis spectrophotometer within the wavelength range 200–750 nm. Characteristic peaks at *λ*_MB_ = 664, *λ*_RhB_ = 554, *λ*_CR_ = 498, *λ*_phenol_ = 269, and *λ*_MO_ = 464.5 nm were monitored to evaluate the extent of organic pollutant degradation. After photodegradation, the catalyst was collected, rinsed with distilled water, and dried. The photocatalytic activity test was repeated for five consecutive cycles with reused samples under identical experimental conditions.

The photodegradation percentage was estimated using Eq. (1):

1 $$\upeta = { }\frac{{C_{0} - C_{t} }}{{C_{0} }}$$

where *C*_*t*_ is the concentration of dye at degradation time *t*, *C*_0_ is the initial concentration of dye, and Ƞ is the degradation efficiency.

The rate constant (*k*) of the degradation reaction was calculated using Eq. (2):

2 $$k{ } = { }\frac{{{\text{ln}}\left( {C_{0} /C_{t} } \right)}}{t}$$

## Supplementary Information

Supplementary Information.
