# Supplementary material for: Selective growth of Ti3+/TiO2/CNT and Ti3+/TiO2/C nanocomposite for enhanced visible-light utilization to degrade organic pollutants by lowering TiO2-bandgap
Source: Sci Rep. 2021 May 4;11:9490. doi: 10.1038/s41598-021-89026-5 (PMC8096813; doi:10.1038/s41598-021-89026-5)
Supplement: Supplementary file 1 — Supplementary Information. [file 41598_2021_89026_MOESM1_ESM.pdf]

Supplementary Materials

Selective growth of $Ti^{3+}/TiO_2/CNT$ and $Ti^{3+}/TiO_2/C$ nanocomposite for enhanced visible-light utilization to degrade organic pollutants by lowering TiO_2-bandgap

Jeasmin Akter[1], Md. Abu Hanif[2], Md. Akherul Islam[2], Kamal Prasad Sapkota[1], Jae Ryang Hahn[1,3]*

[1]*Department of Chemistry, Jeonbuk National University, Jeonju 54896, Korea*

[2]*Department of Bioactive Material Sciences, Jeonbuk National University, Jeonju 54896, Korea*

[3]*Textile Engineering, Chemistry and Science, North Carolina State University 2401 Research Dr., Raleigh, North Carolina 27695, United States*

*Corresponding author

Supplementary Section 1: Characterization methods

Structural identification was conducted by X-ray diffraction (XRD) using Cu Kα radiation (λ = 1.5406 Å). The morphological characterization was carried out by field-emission scanning electron microscopy (FE-SEM) and transmission electron microscopy (TEM) as well as high-resolution TEM (HR-TEM). The FE-SEM and HR-TEM studies were conducted using Hitachi SU8230 and JEOL JEM-2200FS instruments, respectively. A UV–Vis spectrophotometer was used to evaluate the optical properties and estimate the bandgap energy values. The chemical composition was evaluated by energy-dispersive X-ray spectroscopy (EDS). The samples' surface chemical composition and elemental valence state were evaluated by X-ray photoelectron spectroscopy (XPS, NEXSA, Thermo Fisher Scientific, UK). Photoluminescence (PL) analysis was conducted through a spectrophotometer (FP-6500, Jasco). Typical vibrations of the bonds in the nanocomposites were recorded at room temperature by Fourier transform infrared (FTIR) spectroscopy on a Thermo Fisher Scientific Nicolet iS5. For the measurements, the samples were converted into pellets after being mixed with potassium powder. The pore-size distributions and specific surface area of the samples were studied by the Brunauer–Emmett–Teller/Barrett–Joyner–Halenda (BET/BJH) methods (Micromeritics ASAP 2420 V2.09, USA). The MB degradation products were identified using liquid chromatography–mass spectrometry (LC–MS). MS analysis was carried out on an Agilent 6410B instrument (Agilent Technologies, Wilmington, USA). Ions were generated in ionization mode using an electrospray-ionization (ESI) interface. ESI was conducted at +3000 V and at a source temperature of 380°C. The capillary voltage, cone voltage, and source offset were 3 kV, 30 kV, and 30 V, respectively. The gas flow of desolvation and in the cone was set at 650 L/Hr and 150 L/Hr with a nebulizer pressure of 15 bar. A mobile phase composed of 0.1% formic acid in distilled water (buffer A) and 0.1% formic acid in acetonitrile (buffer B) was used to separate the analytes and was pumped into the ESI chamber at a flow rate of 0.5

mL/min for 20 min. The fragmentation potential was set to 110 V, and the interface heater was set to 300°C. Ion detection was carried out in MS full-scan mode.

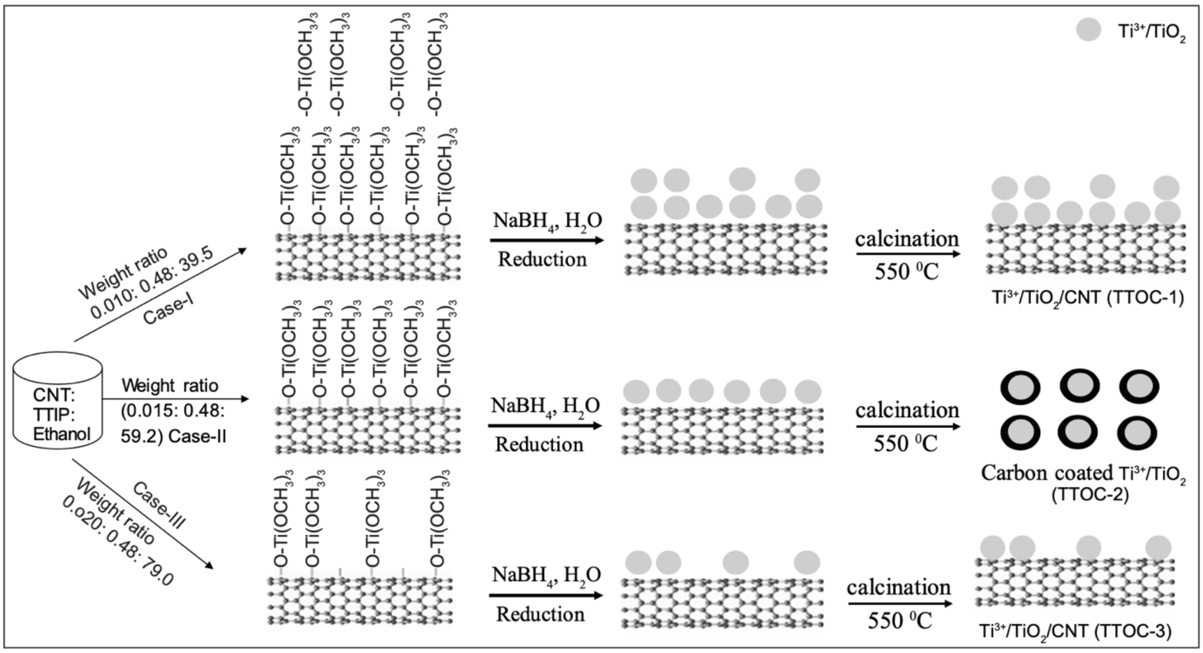

Figure S1. Proposed formation pathways of two different nanocomposite structures: Ti^{3+}/TiO_2/CNT (TTOC-1 and TTOC-3) and Ti^{3+}/TiO_2/C (TTOC-2).

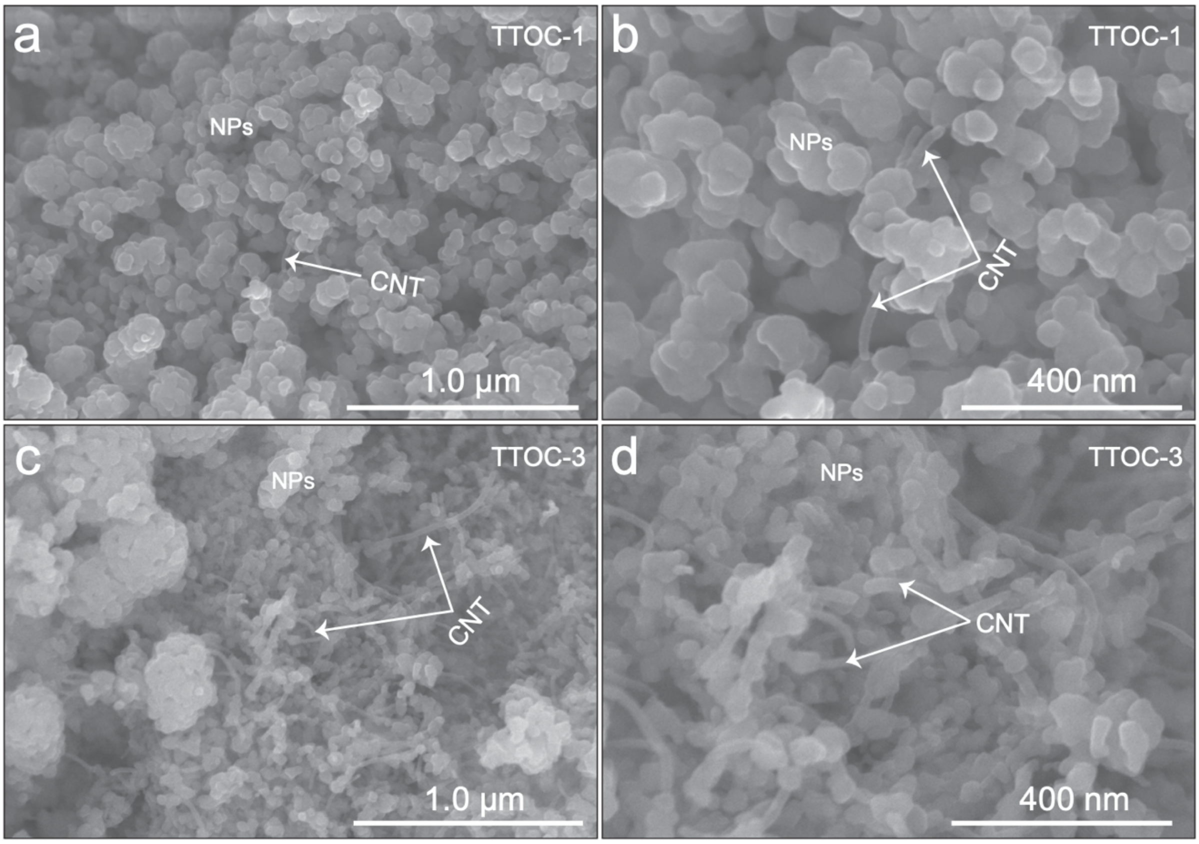

Figure S2. (a) Low-resolution and (b) high-resolution FE-SEM images of the TTOC-1 nanocomposite. (c) Low-resolution and (d) high-resolution FE-SEM images of the TTOC-3 nanocomposite.

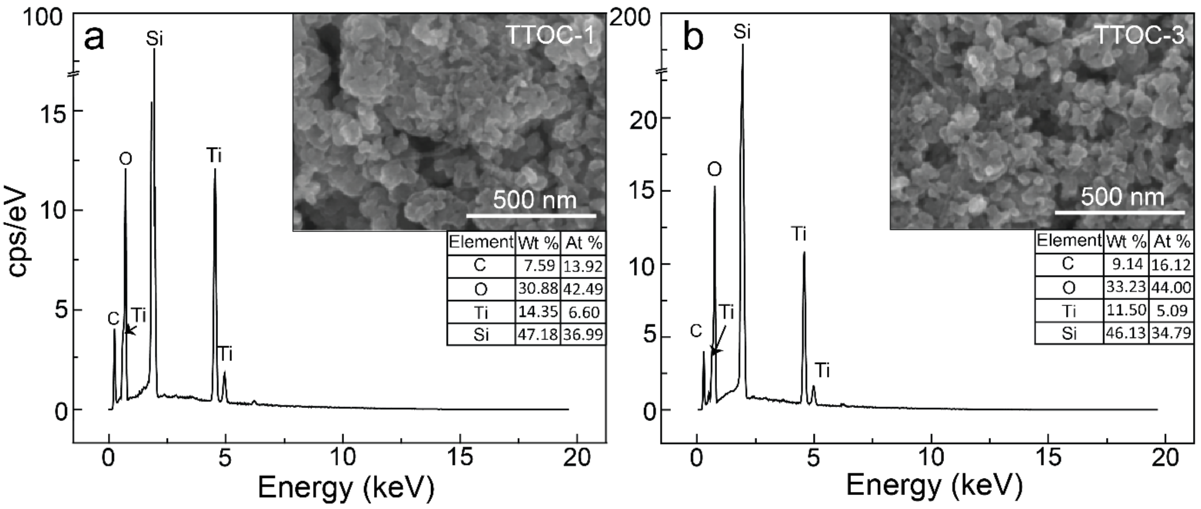

Figure S3. EDS spectra of the (a) TTOC-1 and (b) TTOC-3 nanocomposites. The corresponding regions analyzed by EDS, along with the tabulated results, are shown in insets of each graph.

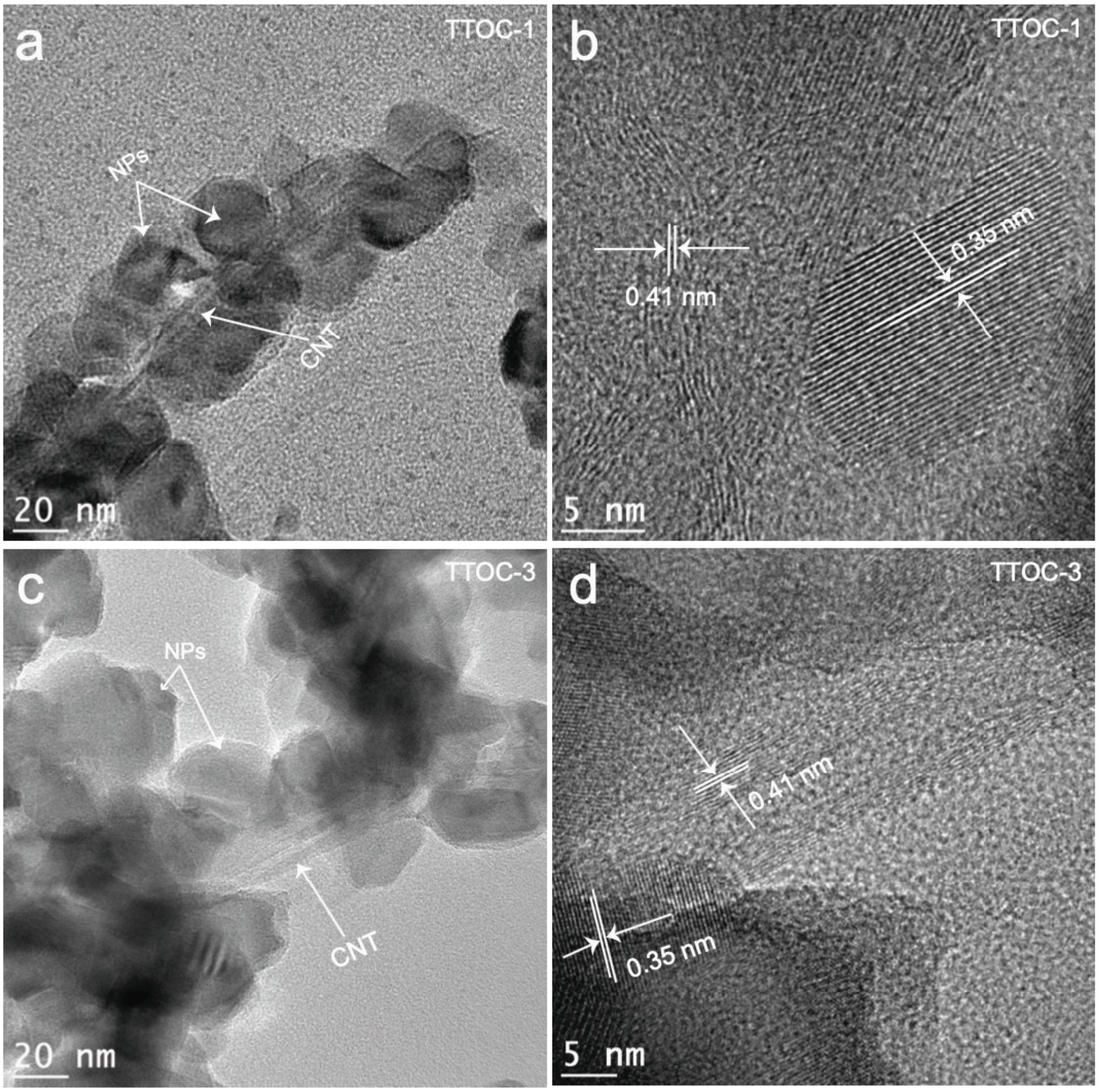

Figure S4. (a) Low-resolution and (b) high-resolution TEM images of the TTOC-1 nanocomposite. (c) Low-resolution and (d) high-resolution TEM images of the TTOC-3 nanocomposite.

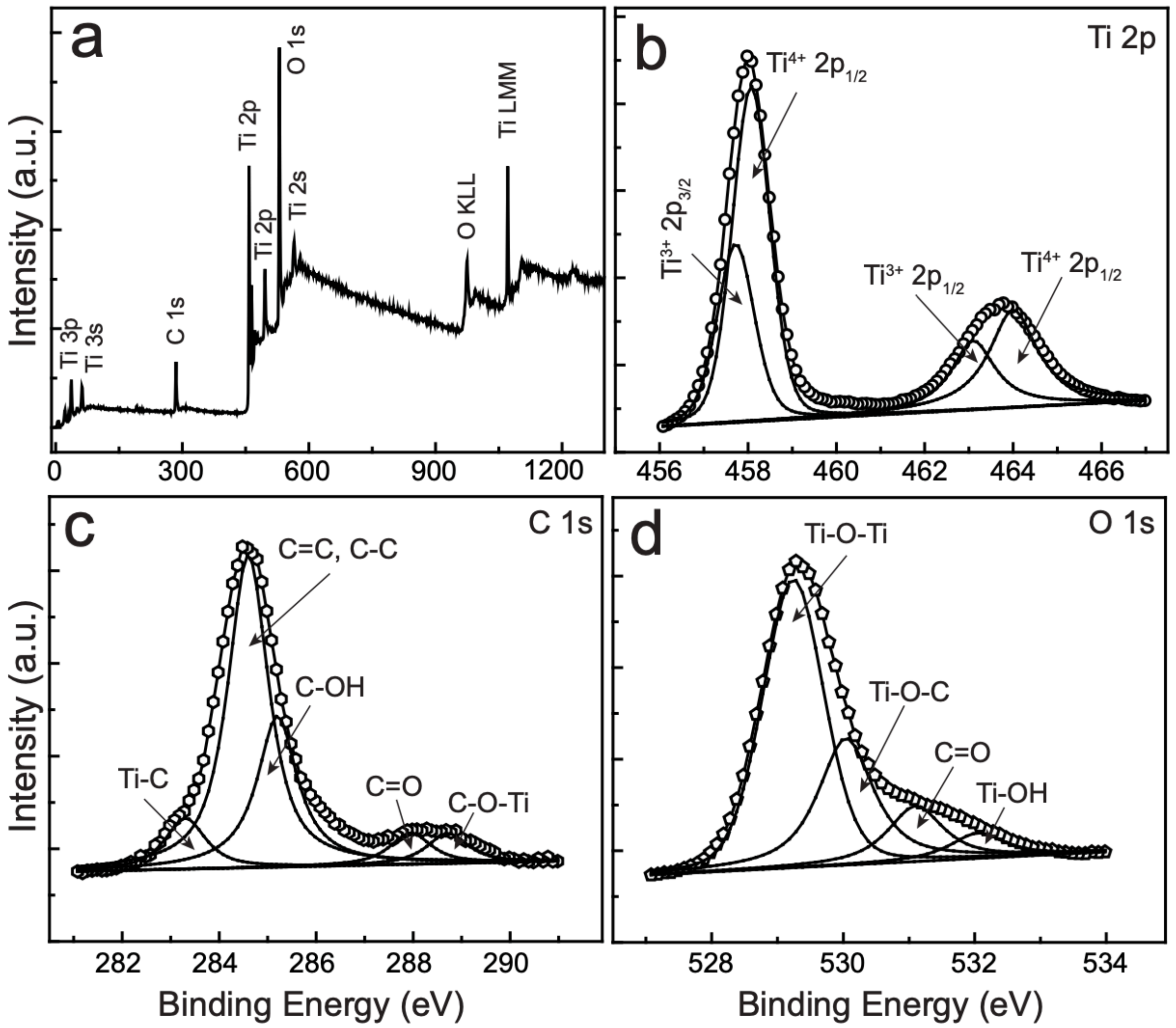

Figure S5. (a) XPS survey spectrum and the (b) Ti-2*p*, (c) C-1*s*, and (d) O-1*s* core-level XPS spectra of the TTOC-1 nanocomposite.

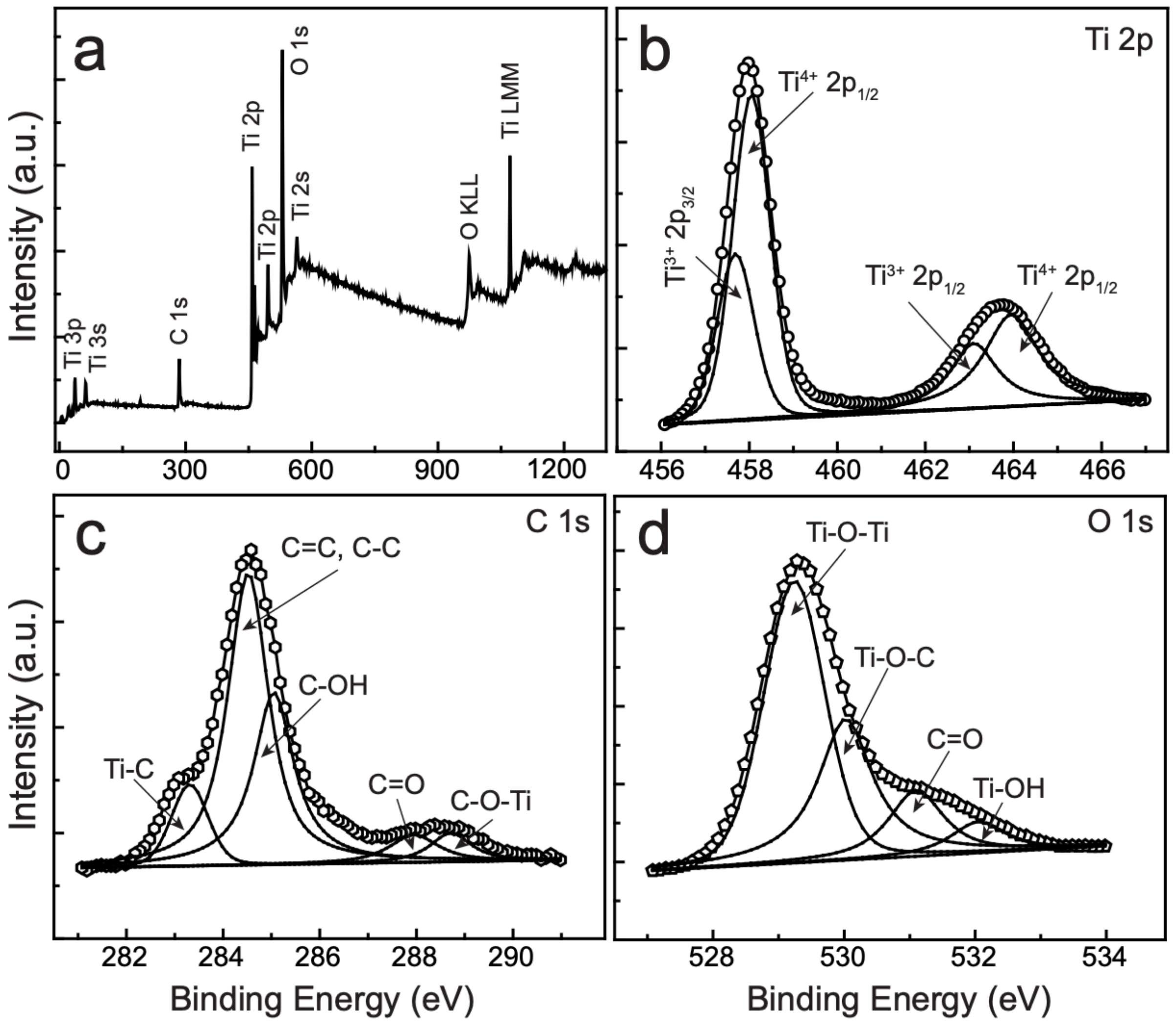

Figure S6. (a) XPS survey spectrum and the (b) Ti-2*p*, (c) C-1*s*, and (d) O-1*s* core-level XPS spectra of the TTOC-3 nanocomposite.

Supplementary Section 2:

The C-1*s* fitting results are presented in Figures 4c, Supplementary Figures 5Sc, and 6Sc for all of the samples, showing the characteristic most intense peak of C–C bonds at 284.64 eV[7]. The C–C bond signal originates from the CNTs (TTOC-1 and TTOC-3) and the carbon layer (TTOC-2) in the nanocomposites. In the spectra of all of the samples, the lower-binding-energy peak at 283.31 eV confirms the metal carbide (Ti–C) bond between the TiO_2 and C[7]. The peaks at 285.23 and 288.74 eV originate from –C–OH and –C–O–Ti bonds, respectively[10, 1]. The –C=O bond peak localized at 287.97 eV is attributed to the presence of a carboxylic group impurity[1]. Figures 4d, Supplementary Figures 5Sd, and 6Sd show the high-resolution O-1*s* spectra of the samples, with four signals. The peak at 529.27 is assigned to the Ti–O–Ti bonds of the TiO_2 lattice[8]. The signal at 530.07 eV is ascribed to the Ti–O–C bonds, i.e., the interaction between the O atoms of TiO_2 and C atoms[8]. The C=O bond peak at 531.13 eV likely arises from carboxylic groups present as an impurity[2]. The signal at 530 eV is attributed to Ti–OH, indicating the formation of –OH groups on the TiO_2[2].

References

1. Hsiao, M. *et al.* Preparation and properties of a graphene reinforced nanocomposite conducting plate. *J. Mater. Chem.* **20,** 8496–8505 (2010).

2. Wang, P. *et al.* The fundamental role and mechanism of reduced graphene oxide in rGO/Pt-TiO_2 nanocomposite for high-performance photocatalytic water splitting. *Appl. Catal. B*. **207,** 335–346 (2017).

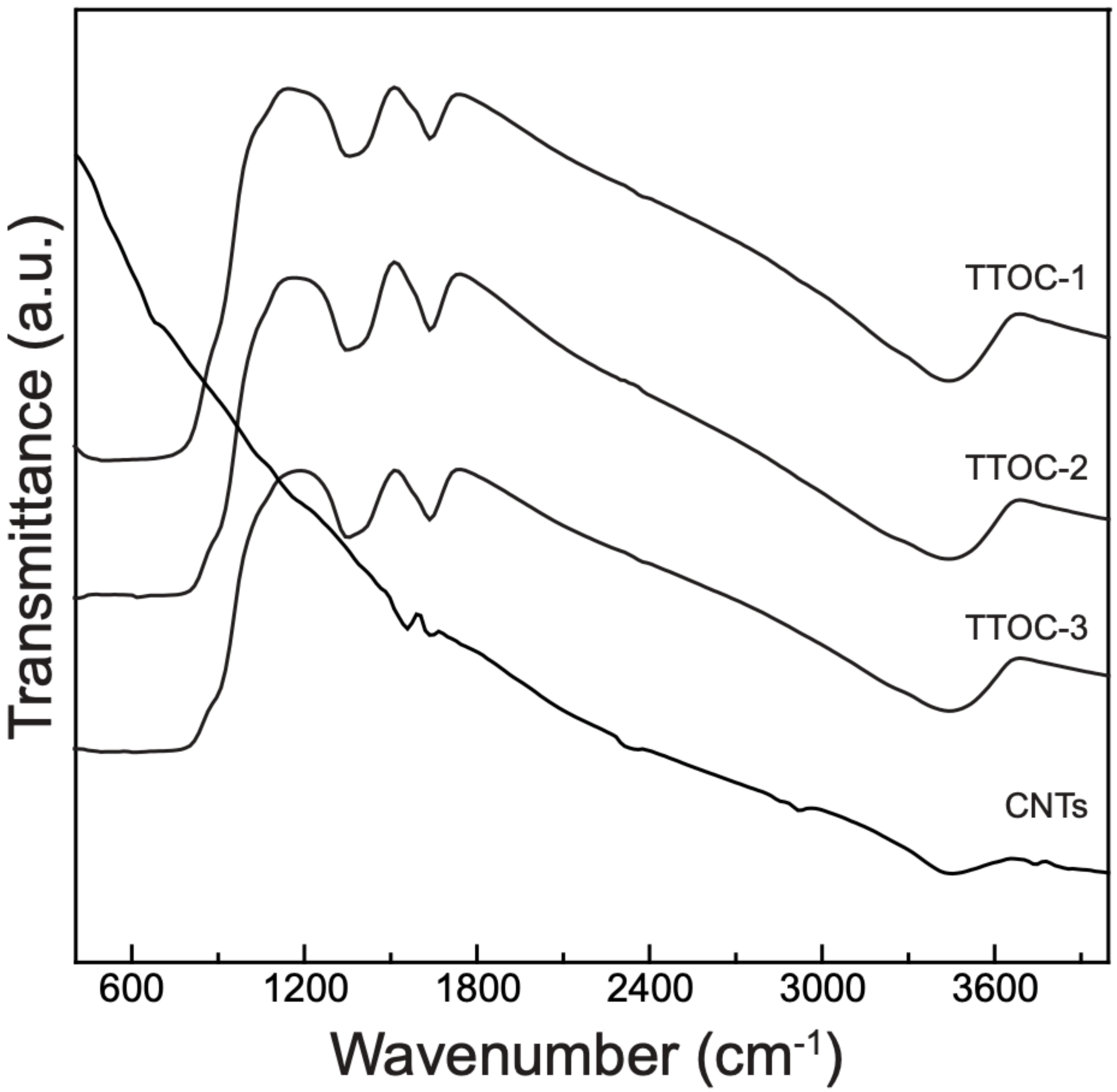

Figure S7. FTIR spectra of as-synthesized nanocomposites (TTOC-1, TTOC-2, and TTOC-3) and pristine CNTs.

Supplementary Section 3:

The functional groups of the nanocomposites were investigated by Fourier transform infrared (FTIR) spectroscopy (Supplementary Figure S7). The FTIR spectra of the bare CNTs and the nanocomposites are illustrated in the range 4000~400 cm^{-1}. The FTIR spectrum of the bare CNTs exhibits two strong peaks at 1554 and 1650 cm^{-1}, which are attributed to stretch mode of C=C bonds in the building units of the graphitic carbon. In the spectra of all of the nanocomposites, peaks are shifted to lower wavelengths at 1344 and 1638 cm^{-1}, indicating strong interaction between the graphitic C and the TiO_2. The spectra of the pristine CNTs and the nanocomposites show a broad peak at 3430 cm^{-1} ascribed to the H–O–H bonding mode of surface-adsorbed H_2O molecules. A broad transmittance peak in the range 800–500 cm^{-1} is due to Ti–O–Ti bond vibrations in TiO_2[11, 12].

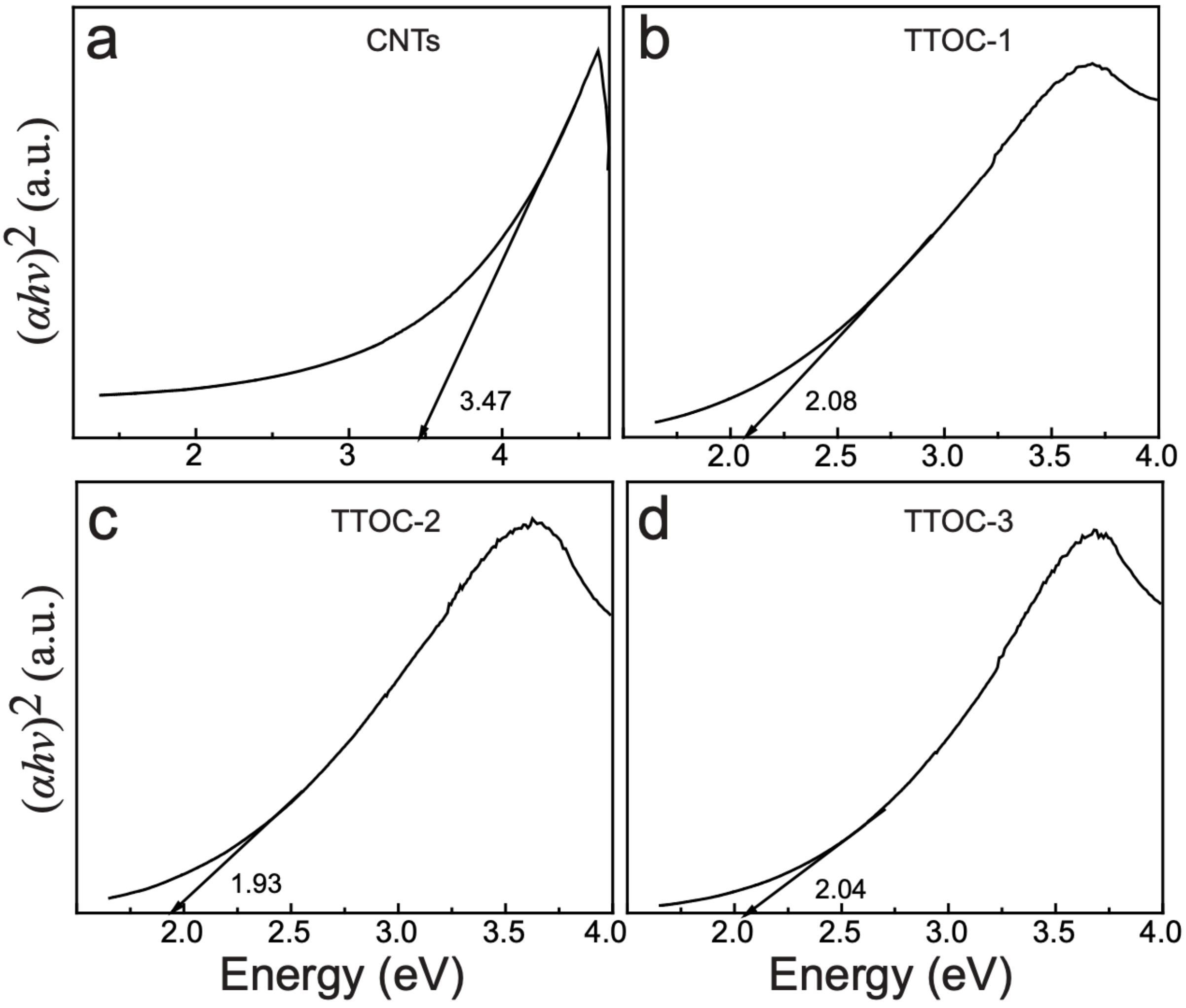

Figure S8. Tauc plots of (a) pristine CNTs and the (b) TTOC-1, (c) TTOC-2, and (d) TTOC-3 nanocomposites.

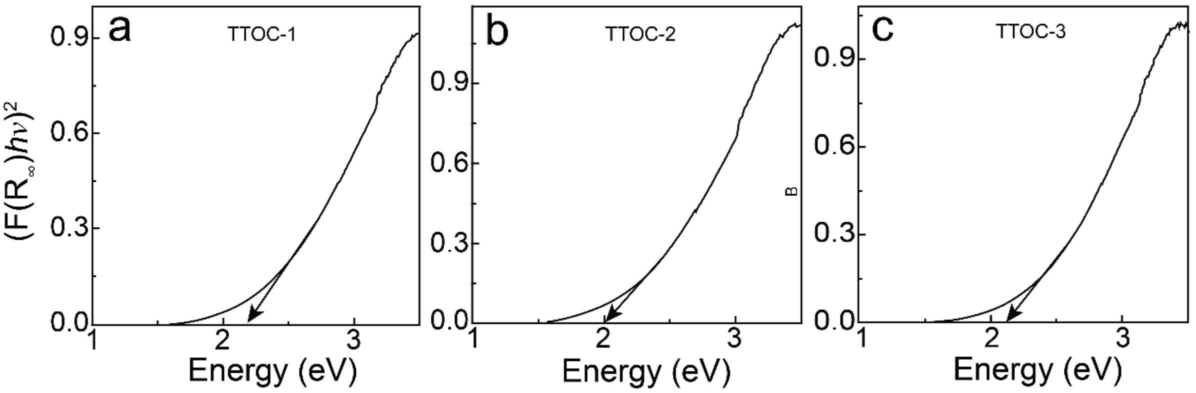

Figure S9. The plot of transformed Kubelka-Munk function vs energy of light for the as-synthesized (a) TTOC-1, (b) TTOC-2, and (c) TTOC-3 nanocomposites, respectively.

Supplementary Section 4:

When the photocatalyst is irradiated, the photoluminescence produces due to photon emission from the (*e*-*h*) pair recombination. At the excitation wavelength of 348 nm, the PL signal of the nanocomposites was found in the range of 593–609 nm (Supplementary Figure S10). The increasing PL intensity follows the order TTOC-2 < TTOC-3 < TTOC-1. With increasing CNTs, the PL intensity generally decreases due to the function of CNT as an electron trapper[23]. This is applicable for TTOC-1 and TTOC-2 nanocomposite, but an exception has occurred for TTOC-2. The vacant site in the nanocomposites causes this trend due to the production of Ti^{3+}. The high Ti^{3+} content demonstrates the presence of a large amount vacant sites[11]. The number of vacant trap sites (BJH and XPS results) also matched well with the PL intensity series. The trapping of electrons in the vacant sites reduces the (*e*-*h*) pairs recombination, resulting in increased photocatalytic activity.

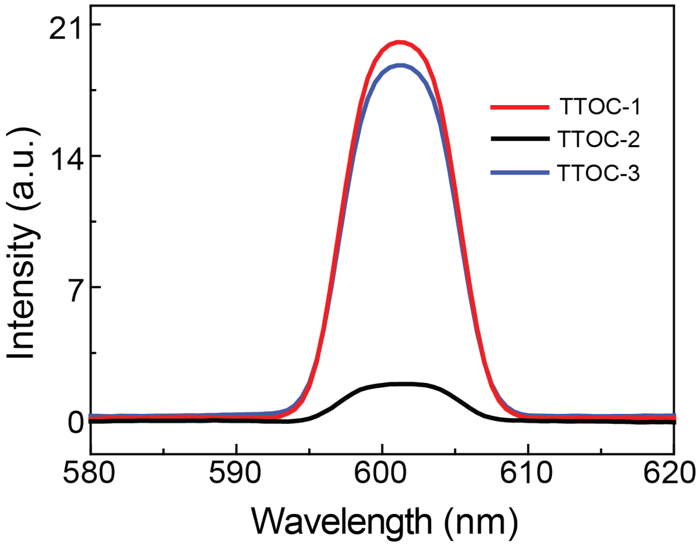

Figure S10. Photoluminescence emission spectra of TTOC-1, TTOC-2, and TTOC-3 nanocomposite photocatalyst.

Supplementary Section 5:

The average surface area was estimated based on the Brunauer–Emmett–Teller (BET) theory. Supplementary Figures S9a–S9d illustrate the adsorption–desorption isotherms of the pristine CNTs and the TTOC-1, TTOC-2, and TTOC-3 nanocomposites, respectively. The corresponding pore-size distribution is presented in the inset of each diagram. The isotherms of the pristine CNTs show an H2 hysteresis loop at relatively low pressures, indicating spontaneous filling of the mesopores. All of the nanocomposites show Type IV adsorption isotherms, where the isotherm reaches a plateau at high relative pressures and the surface is only partially occupied at low pressures.

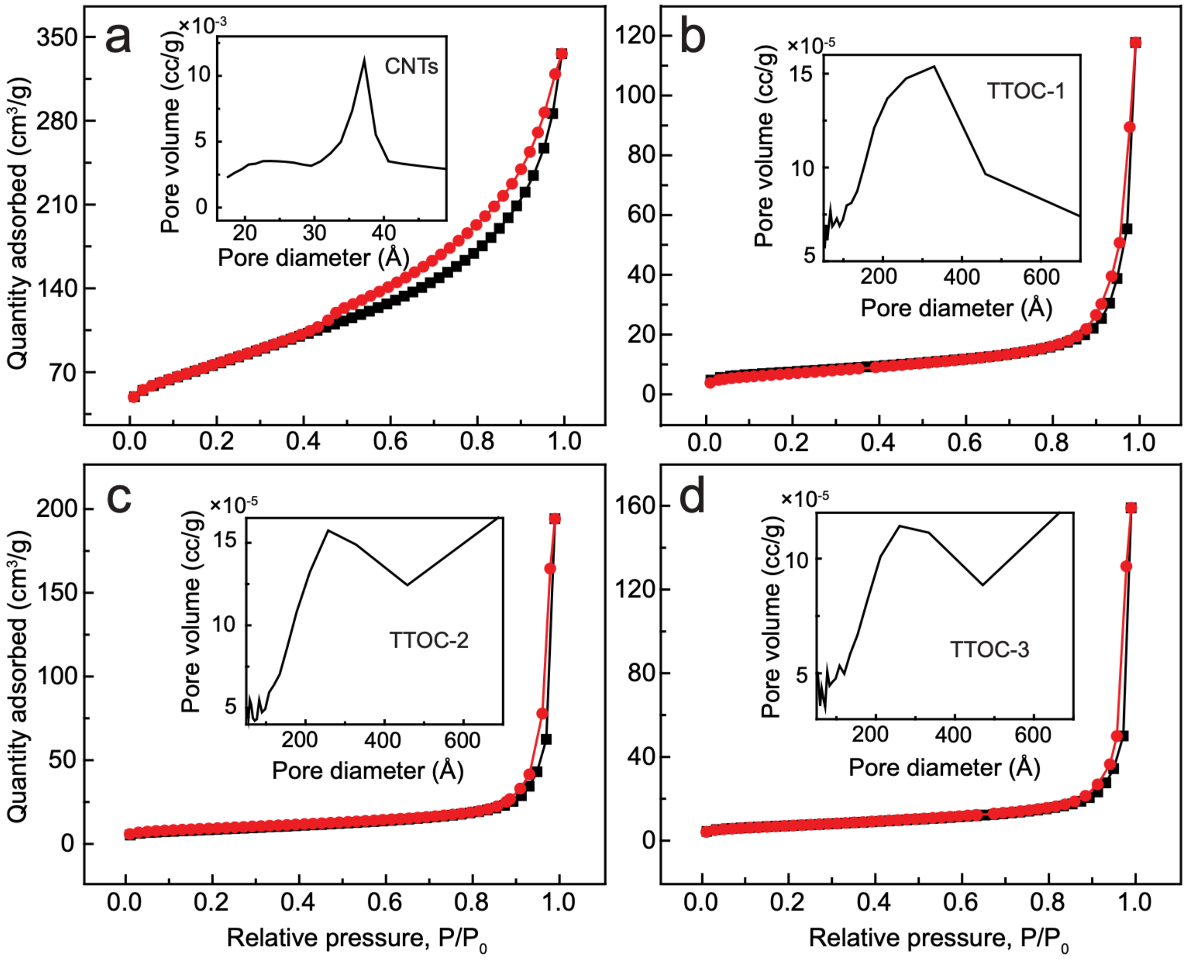

Figure S11. BET adsorption-desorption isotherms and pore size distribution of (a) pristine CNT, (b) TTOC-1, (c) TTOC-2, and (d) TTOC-3 nanocomposites, respectively. Red and Black lines denote desorption and adsorption data, respectively.

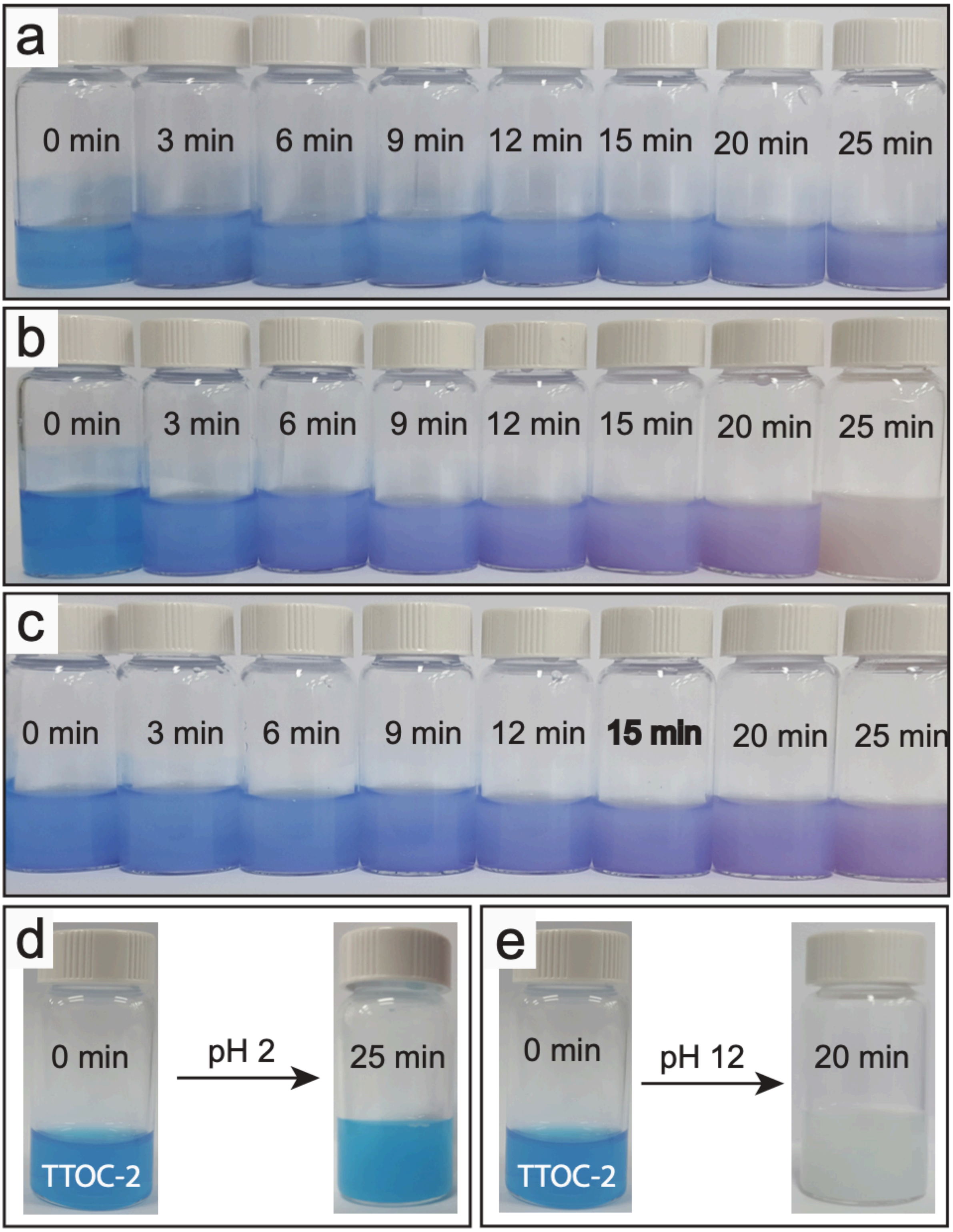

Figure S12. Decoloration of MB solutions containing (a) TTOC-1, (b) TTOC-2, and (c) TTOC-3 nanocomposites. MB degradation using the TTOC-2 nanocomposite at (d) pH 2 and (e) pH 12. The illumination source was visible light.

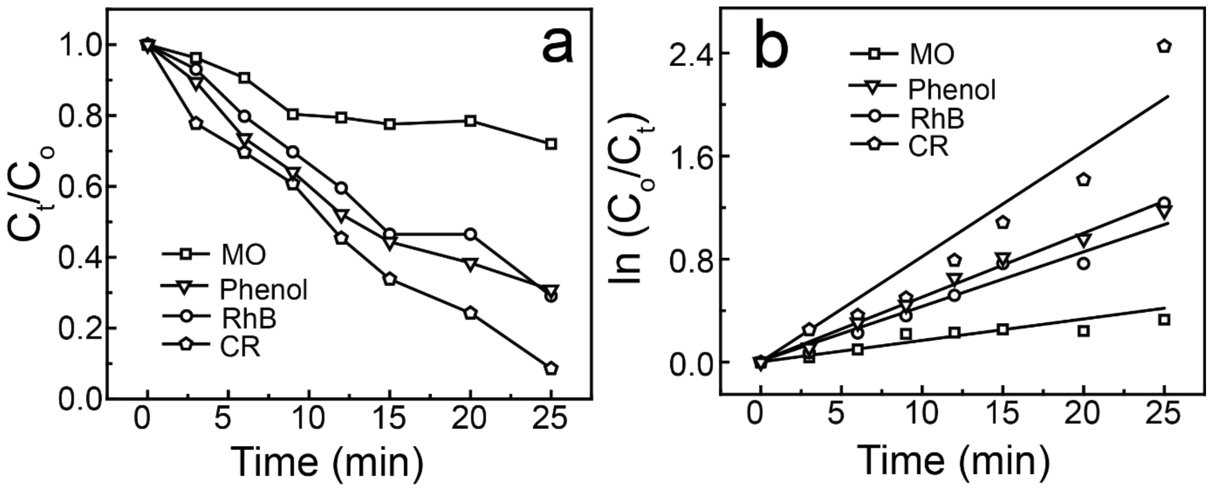

Figure 13. Photocatalytic performance of the TTOC-2 nanocomposite under visible light: (a) degradation ratio over time and (b) first-order kinetics analysis for the degradation reactions of MO, phenol, RhB, and CR.

Table S1. The correlation coefficient (R^2) value of first-order reaction kinetics.

Figure 6b		Figure 13b	
Catalyst	R^2	Pollutant	R^2
TTOC-1	0.9321	CR	0.9217
TTOC-1	0.9253	RhB	0.9880
TTOC-1	0.9313	Phenol	0.9613
CNTs	0.9861	MO	0.9123
WC	0.9677		

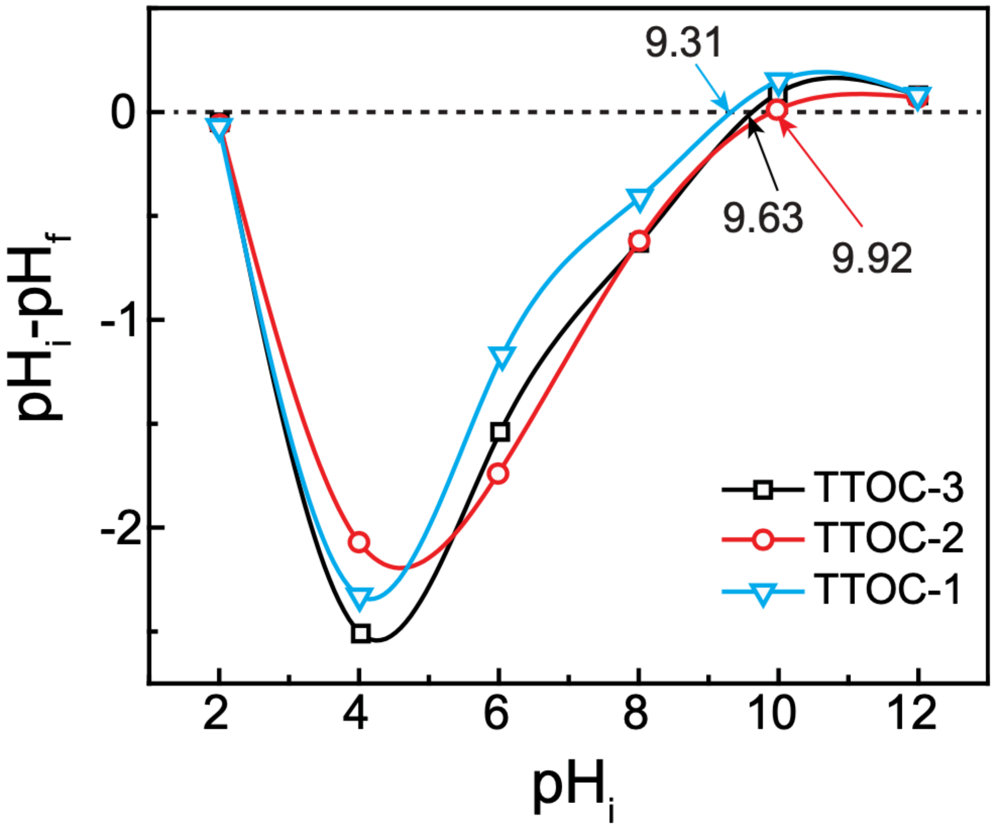

Figure S14. Point of zero charge of the nanocomposites (TTOC-1, TTOC-2, and TTOC-3).

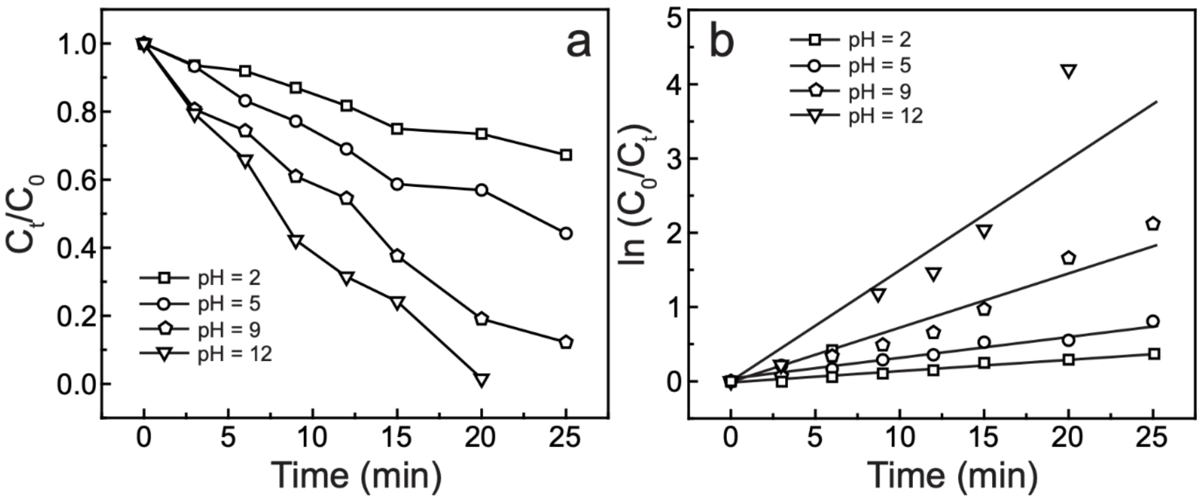

Figure S15. Effect of pH on the MB degradation under visible light using TTOC-2 nanocomposites: (a) photodegradation ratio vs time and (b) first-order kinetics study.

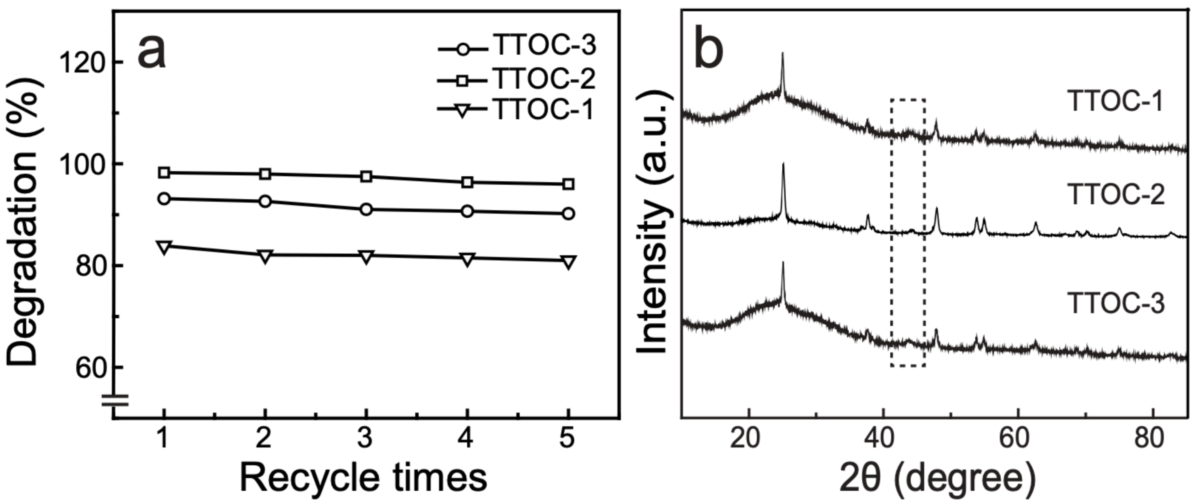

Figure S16. Stability and reusability tests of nanocomposites under visible light: (a) variation of the degradation change over five consecutive cycles and (b) XRD patterns of the photocatalysts after five runs.

Table S2. Comparative studies on the organic pollutant degradation efficiencies of various photocatalysts.

Catalyst	Source of light	Pollutant	Catalyst dose (g)	Dye dose	Degradation (%)	Time (min)	Ref.
MWCNT/TiO_2	Solar	MO	0.1	100 mL, 15 mg/L	87	180	8
TiO_2/Gr/30PW	Tungsten lamp	Phenol	1/L	200 mL, 50 ppm	91	360	9
Ba/ Alg/CMC/TiO_2	Sunlight	CR	1.2/L	30 mL, 4.3×10^{-5} M	91.5	240	10
TiO_2-RGO	Metal-halide lamp	MO	0.05	100 mL, 10 mg/L	87.4	240	16
CNT/TiO_2	Fluorescent lamp	MB	0.05	50 mL, 1×10^{-5} M	58	180	17
Ti^{3+} self-doped TiO_{2-x}	Xenon lamp	MB	0.05	100 mL, 10 mg/L	74	180	20
TiO_2 NRs/CNT	Sunlight	MB	0.02	100 mL, 5 mg/L	97.3	300	21
TiO_2-CNT	UV	MB	0.04	130 mL, 20 ppm	98	180	22
MWCNT/TiO_2	Mercury lamp	MB	0.05	100 mL, 10 mg/L	52	200	23
TiO_2/CNT	Xenon lamp	MB	0.025	50 mL, 10 ppm	38	240	25
Ti^{3+}/TiO_2/CNT	Xenon lamp	MB	0.05	100 mL, 10 mg/L	93	25	Our work
Ti^{3+}/TiO_2/C	Xenon lamp	MB<br>CR<br>RhB<br>Phenol<br>MO	0.05	100 mL, 10 mg/L	98<br>91<br>71<br>69<br>28	25	Our work

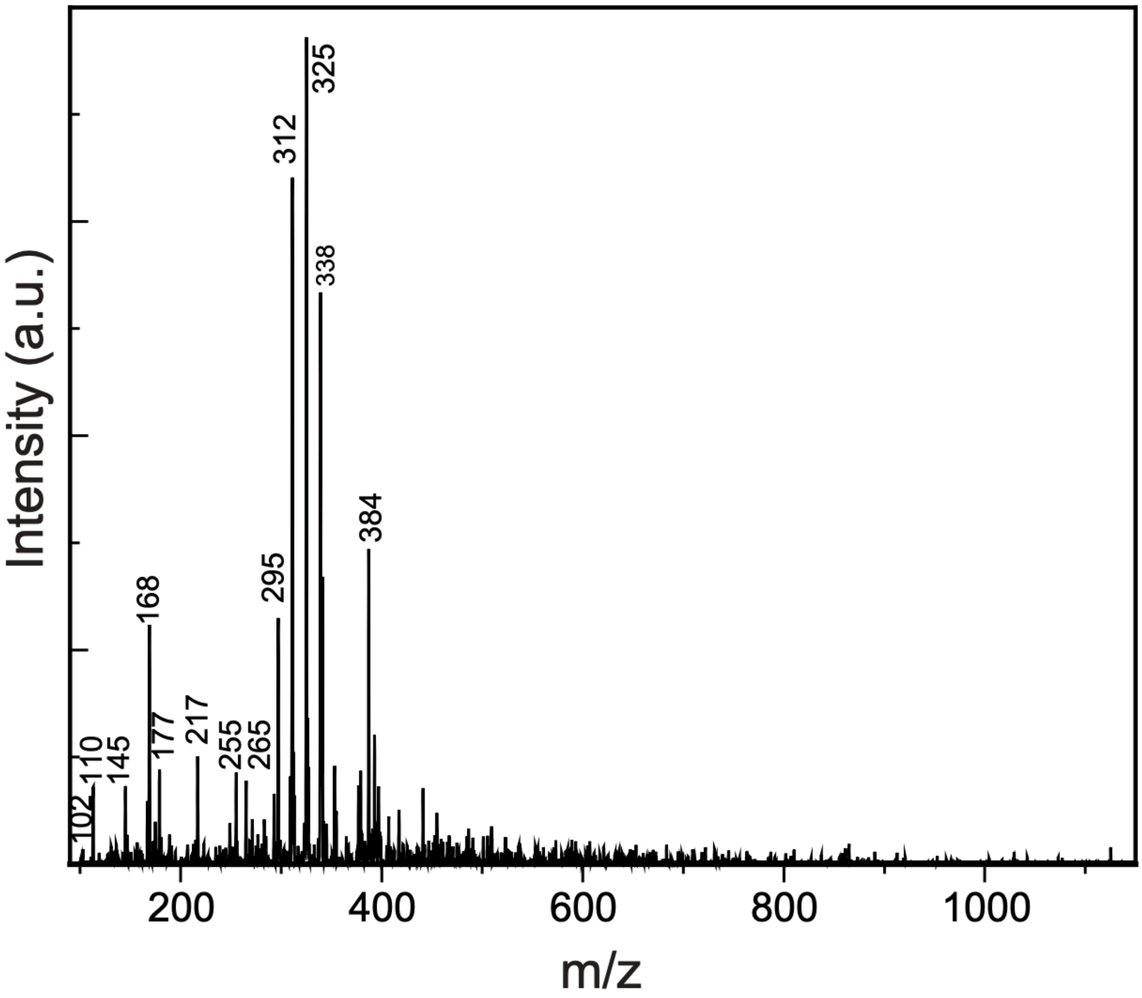

Figure S17. Mass spectra of the aqueous solution of MB after photocatalysis in the presence of the TTOC-2 nanocomposite for 25 min under visible light.

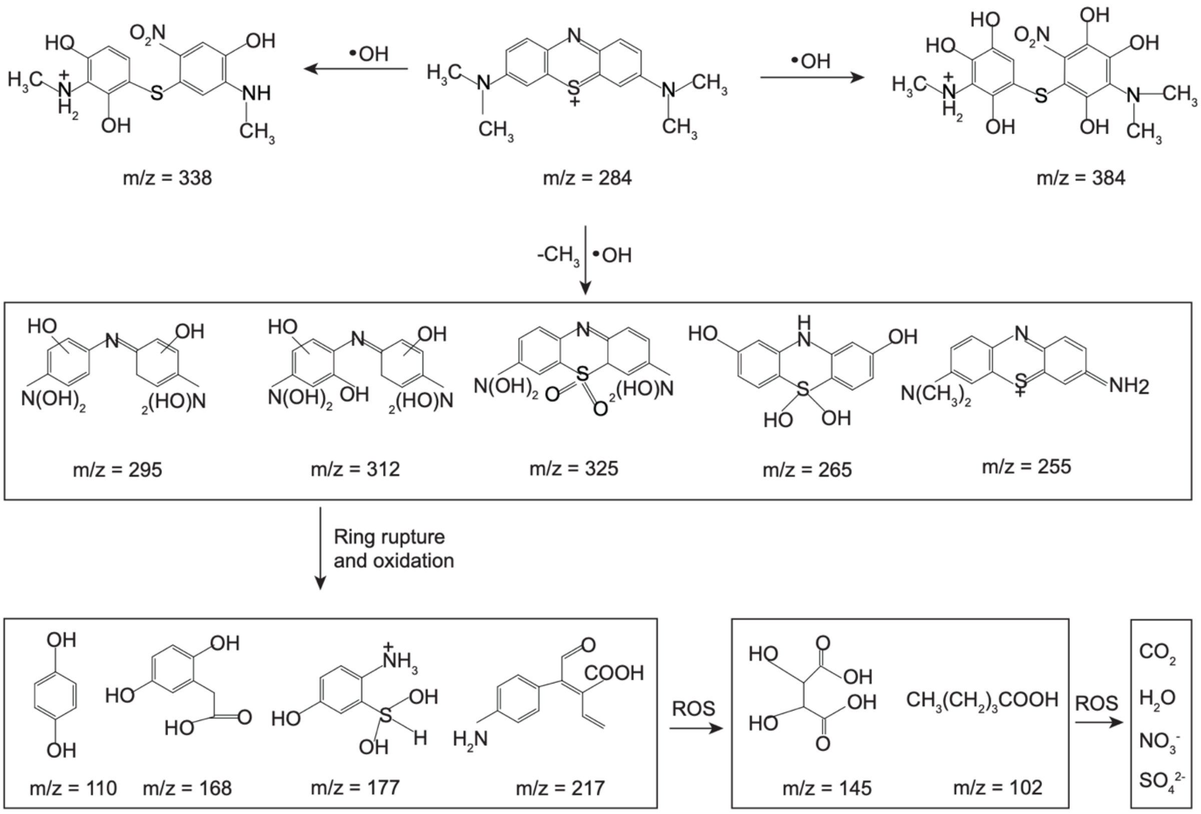

Figure S18. Possible degradation pathway of MB.

Supplementary Section 6: Proposed mechanism of photocatalytic performance

When the energy of incident light is greater than or equal to the bandgap of the catalyst, the VB electrons excite to the CB. The holes in the VB (h^+_{VB}) act as an oxidizing agent; simultaneously, the CB electrons (e^-_{CB}) function as a reducing agent.

$$hv + \text{catalyst} \rightarrow (e^-_{CB} + h^+_{VB})\ \text{catalyst} \quad \text{(S1)}$$

The CB edge potential should be more negative than the acceptor potential, and the VB edge potential should be more positive than the donor potential. On this basis, we calculated the VB and CB potentials using the following formulas:

$$E_{CB} = \chi - E_e - 0.5E_g \quad \text{(S2)}$$

$$E_{VB} = E_{CB} + E_g \quad \text{(S3)}$$

where E_{CB} and E_{VB} represent the CB and VB potentials, respectively, E_e (= 4.5 eV) represents the free electrons' energy on the hydrogen scale, χ indicates the absolute electronegativity, and E_g is the bandgap energy. The proposed reaction mechanism with redox couples and energy band positions is shown in Figure 7. The E_{CB} of the TTOC-1, TTOC-2, and TTOC-3 nanocomposites is positioned at −0.44, −0.36, and −0.42 eV vs normal hydrogen electrode (NHE), respectively. Because the E_{CB} of all the composites is lower than that of $O_2/O_2^{-\bullet}$ (−0.28 eV), superoxide radicals ($O_2^{-\bullet}$) originate from the reduction of dissolved O_2 by the e^-_{CB} (Eq. (S4)):

$$O_2 + e^-_{CB} \rightarrow O_2^{-\bullet} \quad \text{(S4)}$$

The produced $O_2^{-\bullet}$ radicals undergo a series of reactions to produce hydrogen peroxide (H_2O_2) (Eqs. (S5)–(S9)):

$H^+ + O_2^{-\bullet}$	→	$HO_2^\bullet$	(S5)
$HO_2^\bullet + O_2^{-\bullet}$	→	$O_2 + HO_2^-$	(S6)
$HO_2^\bullet + HO_2^\bullet$	→	$O_2 + H_2O_2$	(S7)
$H^+ + HO_2^-$	→	H_2O_2	(S8)
$HO_2^\bullet + HO_2^\bullet$	→	$O_2 + H_2O_2$	(S9)

The H_2O_2 subsequently dissociates into $OH^\bullet$ radicals. The reactive oxygen species ($OH^\bullet$, $O_2^{-\bullet}$, etc.) are the principal oxidizing agents and are responsible for the degradation of the organic pollutant (Eqs. (S10)–(S11)). The E_{VB} values of the TTOC-1, TTOC-2, and TTOC-3 nanocomposites are positioned at 1.64, 1.56, and 1.62 eV vs NHE, respectively. The large h_{VB}^+ potential of all nanocomposites energetically enables the direct hole oxidation of MO, MB, and RhB:

H_2O_2	→	2 $OH^\bullet$	(S10)
Pollutant + (h^+, $O_2^{-\bullet}$, $OH^\bullet$)	→	Degradation Products	(S11)

Supplementary Section 7: Scavenger studies

The degradation of MB via TTOC-2 photocatalyst was tested using BQ, IPA, and KI as a scavenger of $O_2^{-\bullet}$, $OH^{\bullet}$, and h^+, respectively. The experimental condition was the same, and the amount of trapping agent was 0.5 mmol/L. As shown in Figure S19, all the trapping agents reduce the catalyst activity to a certain extent than the reaction with no scavengers (NS). The order of active species was $O_2^{-\bullet} > h^+ > OH^{\bullet}$, based on the effectiveness. Since the photocatalytic activity was significantly influenced by the addition of BQ, which suggests $O_2^{-\bullet}$ plays a dominant role in the degradation system. The h^+ was also an important active species in the photocatalytic reaction. Although the role of $OH^{\bullet}$ is not so crucial compared to $O_2^{-\bullet}$ and h^+ but helps to improve the reaction rate. All the active species ($O_2^{-\bullet}$, $OH^{\bullet}$, h^+) were generated and involved in the photodegradation process. The outcomes well matched with the proposed photocatalytic reaction mechanism using as-synthesized nanocomposites. ROS plays a significant role in the enhanced rate of photocatalytic reaction.

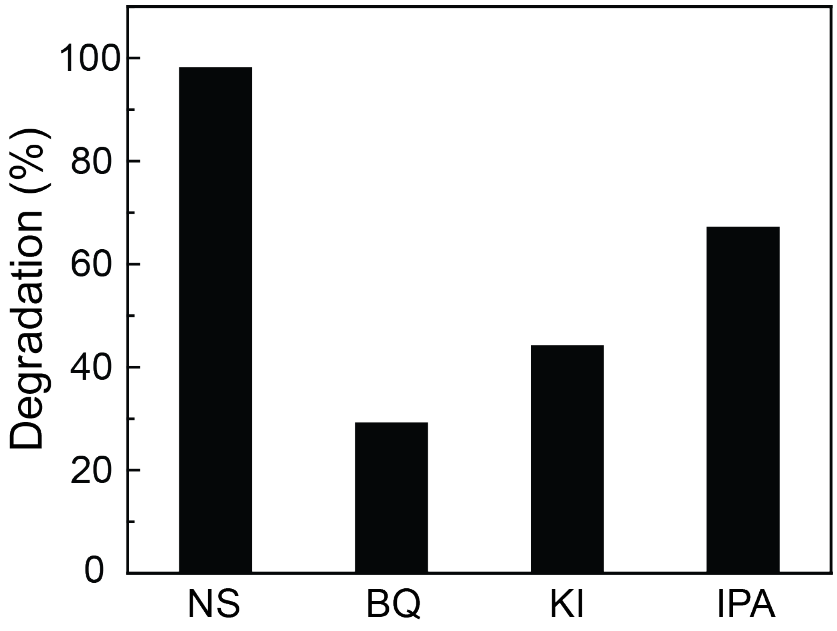

Figure S19. MB degradation efficiency of TTOC-2 in the presence of different scavengers within 25 min under VL.
